# Evaluation of a complex intervention for prisoners with common mental health problems, near to and after release: the Engager randomised controlled trial

**DOI:** 10.1192/bjp.2022.93

**Published:** 2023-01

**Authors:** Richard Byng, Tim Kirkpatrick, Charlotte Lennox, Fiona C. Warren, Rob Anderson, Sarah Louise Brand, Lynne Callaghan, Lauren Carroll, Graham Durcan, Laura Gill, Sara Goodier, Jonathan Graham, Rebecca Greer, Mark Haddad, Tirril Harris, William Henley, Rachael Hunter, Sarah Leonard, Mike Maguire, Susan Michie, Christabel Owens, Mark Pearson, Cath Quinn, Sarah Rybczynska-Bunt, Caroline Stevenson, Amy Stewart, Alex Stirzaker, Roxanne Todd, Florian Walter, Lauren Weston, Nat Wright, Rod S. Taylor, Jenny Shaw

**Affiliations:** Community and Primary Care Research Group, University of Plymouth, UK; Division of Psychology and Mental Health, University of Manchester, UK; College of Medicine & Health, University of Exeter, UK; Centre for Mental Health, South Bank Technopark, London, UK; City, University of London, UK; Kings College London, UK; Research Department of Primary Care and Population Health, University College London, Royal Free Medical School, UK; Centre for Criminology, University of South Wales, UK; Wolfson Palliative Care Research Centre, Hull York Medical School, Faculty of Health Sciences, University of Hull, UK; South West Mental Health Clinical Network, NHS England, UK; University of Leeds, UK; MRC/CSO Social and Public Health Sciences Unit & Robertson Centre for Biostatistics, Institute of Health and Well Being, University of Glasgow, UK; Division of Psychology and Mental Health, University of Manchester, UK and Greater Manchester Manchester Health NHS Foundation Trust, UK

**Keywords:** Offender, common mental health problem, complex intervention, randomised controlled trial, prison

## Abstract

**Background:**

Many male prisoners have significant mental health problems, including anxiety and depression. High proportions struggle with homelessness and substance misuse.

**Aims:**

This study aims to evaluate whether the Engager intervention improves mental health outcomes following release.

**Method:**

The design is a parallel randomised superiority trial that was conducted in the North West and South West of England (ISRCTN11707331). Men serving a prison sentence of 2 years or less were individually allocated 1:1 to either the intervention (Engager plus usual care) or usual care alone. Engager included psychological and practical support in prison, on release and for 3–5 months in the community. The primary outcome was the Clinical Outcomes in Routine Evaluation Outcome Measure (CORE-OM), 6 months after release. Primary analysis compared groups based on intention-to-treat (ITT).

**Results:**

In total, 280 men were randomised out of the 396 who were potentially eligible and agreed to participate; 105 did not meet the mental health inclusion criteria. There was no mean difference in the ITT complete case analysis between groups (92 in each arm) for change in the CORE-OM score (1.1, 95% CI –1.1 to 3.2, *P* = 0.325) or secondary analyses. There were no consistent clinically significant between-group differences for secondary outcomes. Full delivery was not achieved, with 77% (108/140) receiving community-based contact.

**Conclusions:**

Engager is the first trial of a collaborative care intervention adapted for prison leavers. The intervention was not shown to be effective using standard outcome measures. Further testing of different support strategies for prison with mental health problems is needed.

## Needs of prison leavers

Individuals in contact with the criminal justice system internationally have high levels of psychiatric problems,^1^ co-occurring substance misuse, unstable housing and social adversity, and are known to receive very little mental health treatment once out of prison.^2^ Prevalence rates for prisoners can vary greatly because of the way problems are assessed, but rates of over 50% for depression and 25% for anxiety are not uncommon.^3^ Rates of personality disorder and post-traumatic stress disorder (PTSD) are also high and significant proportions also having a range of cognitive and behavioural problems linked to autism, attention-deficit hyperactivity disorder, intellectual difficulties/disabilities (also known as learning disabilities in UK health services) and/or traumatic brain injury.^4^ For those coming in and out of prison repeatedly, a similar picture of comorbidity exists, with high suicide rates and frequent transitions.^5^ Very little healthcare is accessed beyond substance misuse services and contact with general practice and emergency departments.^2,6^ This high need but low service use provides a rationale for developing and evaluating interventions specifically for prison leavers with mental health problems and UK policy supports such continuity.^7^

## Rationale for intervening

Although providing theoretical grounds for potential health gain, previous research into such interventions is very limited.^8^ Randomised controlled trials (RCTs) in a prison context can be complex because of organisational challenges, problems in maintaining masking, poor follow-up rates and selecting appropriate outcome measures.^1,9–11^ Interventions such as collaborative care (based on a chronic disease management model) have been shown to be beneficial for those with depression^12^ but have not been tested in RCTs with prison leavers. Elements of collaborative care have the potential to ensure continuity over time, coordination between teams, as well as support for self-care. There has been some focus on delivery of ‘through-the-prison-gate’ interventions,^13^ providing day of release contact to ensure links to essential services to address homelessness and substance misuse. Critical time intervention (CTI), a structured, time-limited form of case management has been adapted for a prison population with psychosis, been successfully evaluated and demonstrated increased engagement at 6 weeks post release from prison.^14^ Theoretically, individuals could benefit from evidence-based therapies for different conditions. Those with problems associated with personality disorder could be helped with dialectical behavioural therapy, mentalisation-based treatment, or structured clinical management; and many would benefit from a range of social interventions and joined-up substance misuse care, if required. To date, to the best of our knowledge, there are no high-quality RCTs of any specific mental health interventions for prison leavers and no rigorous studies attempting to bring all these aspects together.

## The Engager programme

The Engager programme built on earlier work demonstrating low levels of contact and a lack of trust,^15^ poorly coordinated services, and missed opportunities to start care in prison and that continue on release.^2^ Feasibility work demonstrated the ability to recruit in prison and follow-up individuals in the community.^10,11^ A theoretically informed intervention (‘Engager’) was developed combining therapeutic work and organisational support through mixed methods including a realist review,^16^ case studies,^17^ focus groups, peer-research involvement and adaptation in a formative evaluation during the pilot trial.^18^ This paper reports the results of the RCT with the aim of investigating the effect of the Engager intervention plus usual care, compared with usual care alone, on psychological and social outcomes in men with common mental health problems in prison and in the months following release from prison.

## Method

The trial was conducted and reported in accordance with the Consolidated Standards of Reporting Trials (CONSORT) guidelines; a copy of the CONSORT checklist is in the Supplementary material (available at https://doi.org/10.1192/bjp.2022.93) and the detailed methods described in the full trial protocol.^19^

### Study design and participants

Engager was a two group parallel randomised superiority trial (ISRCTN11707331) with allocation to either the Engager intervention plus usual care (intervention group) or usual care alone (control group) across two investigation centres (prisons and nearby localities in South West and North West of England).

Inclusion criteria were: men serving a prison sentence of 2 years or less; who had between 4 and 20 weeks remaining until release; who were being released to the geographical area of the study; who were willing to engage with treatment services and research procedures; and who were identified as likely to have depression, anxiety or PTSD currently or following release. Details of how each inclusion or exclusion criterion was assessed are described in the Supplementary material.

Exclusion criteria were: men awaiting trial (remand); with serious and enduring mental disorder and/or on the case-load of the prison in-reach team; men who were under the offender personality disorder pathway service; with active suicidal intent; who presented a serious risk of harm to the researchers or intervention practitioners; and/or those who were unable to provide informed consent. Informed consent was obtained from all participants.

The trial was overseen by an independent trial steering committee. The authors assert that all procedures contributing to this work comply with the ethical standards of the relevant national and institutional committees on human experimentation and with the Helsinki Declaration of 1975, as revised in 2008. All procedures involving patients were approved by the UK National Health Service, Wales Research Ethics Committee 3 (ref: 15/WA/0314), and the National Research Committee of Her Majesty's Prison and Probation Service.

### Randomisation and masking

Participants were individually randomised on a 1:1 ratio. Randomisation was achieved via a web-based system, using a block design stratified by prison cluster to ensure balance between the two treatment arms at each centre. Randomisation numbers were assigned in strict sequence with participants being assigned the next randomisation number in the sequence at the point of randomisation. The trial manager was notified of allocation by email and the research assistants delivered a letter to the participant in prison to inform them of the allocation. Owing to the nature of the intervention, participants were aware of their allocation. In addition, following the challenges observed in the pilot trial,^11^ the research assistants collecting data were not masked to allocation. A statistician masked to group allocation undertook the primary analysis.

### Intervention and usual care

Engager was designed to engage individuals with common mental health problems, developing a pathway of care for release and resettlement in the community. It was a manualised, person-centred intervention aiming to address mental health needs as well as to support wider issues such as accommodation, education, social relationships and money management.

The Engager practitioners were informed of each participant's assignment to Engager on the day of randomisation. The intervention was delivered in prison between 4 and 16 weeks pre-release and for up to 20 weeks post release. Experienced support workers and a supervisor with experience of psychological therapy delivered Engager.

The practitioner and participant developed a shared understanding of the participant's needs and goals, recognising the links between emotion, thinking, behaviour and social outcomes. A plan was developed, based on agreed goals and including liaison with relevant agencies and the participant's social networks. A mentalisation-informed approach underpinned all elements of the intervention.^20^ Use of existing practitioner skills was also key to intervention delivery.

Engagement was maintained before release and all-day support was given on the release day when required. Following release, the practitioner provided support for the participant through flexible face-to-face and telephone contact. They continued to work with the participant and any relevant organisations to help them achieve their goals, while encouraging the participant to take responsibility for self-care. The practitioner also prepared the participant for the end of the intervention, while liaising with relevant community organisations regarding continuity of care.

Participants assigned to usual care had access to all usual services for example primary care, secondary mental health, substance misuse services, criminal justice and any other third-sector organisations in the standard way. A detailed description of usual care can be found in Supplementary material.

### Outcomes

The primary outcome was psychological distress using the Clinical Outcomes in Routine Evaluation Outcome Measure (CORE-OM) assessed at 6 months after release from prison. Outcome measures had been selected using a consensus process; the CORE-OM and CAN–FOR (Camberwell Assessment of Need – Forensic Version) were equally ranked as contenders for primary outcome measure, with the former selected on the basis of psychometric properties. The CORE-OM is not diagnosis specific, suiting the population, is well recognised, accessible, widely used and also included questions related to safety and social problems.

Secondary outcomes comprised: (a) CAN–FOR; (b) Treatment Outcomes Profile; (c) Leeds Dependence Questionnaire; (d) Brief Inspire; (e) EuroQol EQ-5D 5 Level and utility tariff; (f) ICEpop CAPability Adult and related tariff; (g) Intermediate Outcomes Measurement Instrument; (h) an adapted version of the Client Service Receipt Inventory; and (i) accommodation. The reoffending rate was planned as a further outcome, and permission obtained but data was not available at the end of the trial.

All participants were assessed at six time points: baseline (before randomisation), during the week before release from prison (pre-release), and then at 1, 3, 6- and 12-months post release from prison. An overview of all of the measures used, including associated references can be found in the Supplementary material. Serious adverse events were recorded and monitored throughout the study.

A mixed-methods process evaluation was conducted in parallel with the trial. It covered assessment of: intervention fidelity; barriers and facilitators to implementation; and how the components of the intervention worked individually and as a whole. A separate cost-utility and cost-consequence analysis was carried out. This paper reports the quantitative aspects of fidelity assessed by the numbers of contacts before and after release.

### Statistical analysis

To detect a change in CORE-OM score of 3.5, equivalent to a small-to-moderate effect with an alpha threshold of 0.05 and 90% power, and assuming a standard deviation of 7.5, 140 participants were estimated to be needed in each group, allowing for 30% attrition. All analyses were prespecified in a statistical analysis plan that was reviewed by the trial steering committee (See Supplementary material for the detailed statistical analysis plan).

The primary analysis for primary and secondary outcomes was based on the intention-to-treat (ITT) principle using observed data for participants at 6-month follow-up. Analysis was performed using regression modelling, with linear regression used for continuous outcomes and logistic regression for binary outcomes. We report between-group differences (Engager plus usual care versus usual care only) at 6- and 12-month follow-up with 95% CIs and *P*-values.

Secondary analyses included a per protocol analysis (including only participants in the intervention arm who received at least two sessions in prison and eight sessions in the community, the minimum considered necessary to generate an effect), using complier average causal effect (CACE) analysis, and a repeated-measures analysis including all observed data across time points, using a mixed-effects regression model with a random effect on participant.

The issue of missing data was addressed by comparing the participant characteristics of the baseline cohort with those of participants who had observed CORE-OM data at 6- and 12-month follow-up, to descriptively evaluate whether there were any substantive differences in characteristics of participants with observed data across time points. For the primary and secondary outcome variables, we performed a multiple imputation algorithm using chained equations to impute data for those participants who had a missing observation at a specific time point. A further ITT analysis was then performed with observed and imputed data. Differential treatment effects were investigated by addition of an interaction term to the model (ITT, observed data) between intervention and participant baseline characteristic (site, trauma, personality disorder, unstable versus stable accommodation, alcohol problem, substance use); a separate model was performed for each interaction.

All analyses were adjusted for study site and baseline scores (for continuous outcome measures). Analyses were adjusted for any participant characteristics that were unbalanced and performed by an analyst masked to group allocation. Collection and cleaning of the 12-month follow-up data was completed before allocation was revealed, although analysis of the 12-month data was performed unmasked.

## Results

**Fig. 1:**
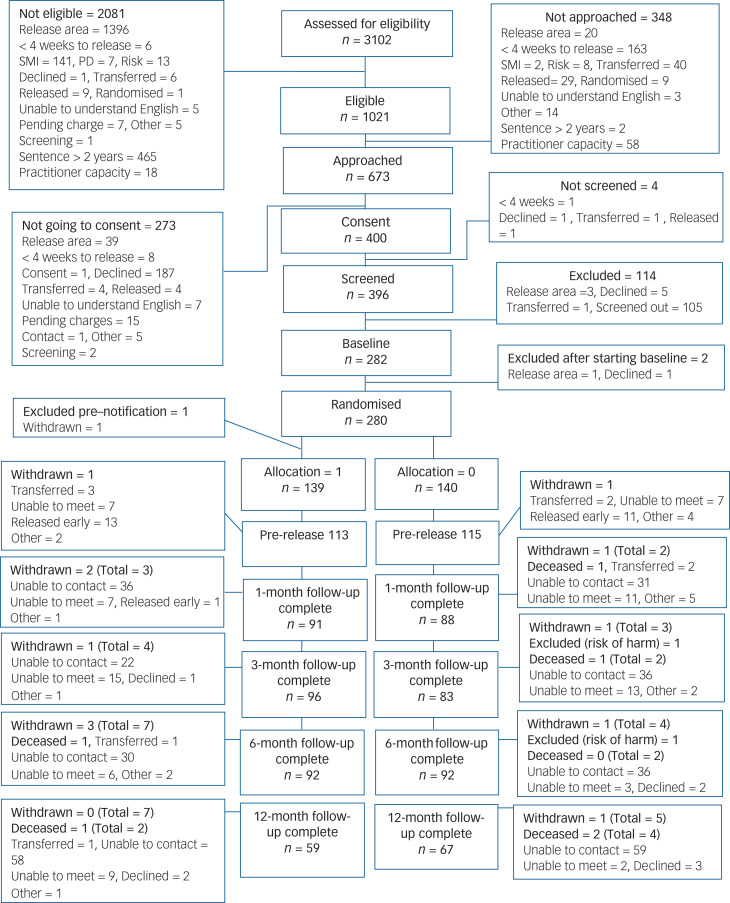
CONSORT diagram. SMI, severe mental illness; PD, personality disorder.

Between 21 January 2016 and 3 October 2017, we assessed 3102 individuals for eligibility according to release time and location. Of the 589 we approached who were eligible, 187 declined involvement, with 402 (68%) agreeing to be assessed. In total, 396 were screened for common mental health conditions, of whom 105 did not meet the clinical criteria, 6 declined and a further 5 had become ineligible (e.g. because of change in release area). Overall, 73% (291/396) of those screened were eligible. Of the 280 remaining after further exclusions, 206 (74%) scored >10 on the 9-item Patient Health Questionnaire, 188 (67%) scored >10 on the 7-item Generalised Anxiety Disorder and 148 (53%) scored ≥3 on the PTSD screening tool. We randomly assigned the 280 participants, 140 to the Engager intervention and 140 to usual care. One person in the intervention group withdrew before notified of allocation. A further 11 participants withdrew before the 12-month follow-up and an additional 4 intervention and 2 control participants died during this time period (Fig. 1 shows the consort diagram).

Baseline characteristics of the trial participants are shown in Table 1. The average age of participants was 34 years and most (94%) were White. Under half were in stable accommodation and just over two-thirds had been unemployed prior to incarceration. Just over half of the sample were in receipt of benefits as their main source of income and 14% (*n* = 33) had no declared source of income. Four-fifths of the sample were single (*n* = 227; 81%) and about a fifth had rare or no contact with parents or siblings, suggesting limited close relationships. Health problems were common in this sample. About two-fifths had physical health problems. Rates of previous head injury, drug and alcohol problems and self-harm were high, with 56% (*n* = 136) of the sample having a history of self-harm.

**Table 1:** Participant demographic characteristics and prison history at baseline

	All participants	
Characteristic	Engager (*n* = 140)	Usual care (*n* = 140)
Age, years: mean (s.d.) *n*; median (range)	34.3 (11⋅4), 140; 32 (18–65)	34.8 (9.9), 139; 33 (19–62)
Ethnic group, *n* (%)	(*N* = 138)	(*N* = 139)
White	128 (93)	133 (96)
Other	10 (7)	6 (4)
Pre-prison accommodation, *n* (%)		
Stable	56 (40)	73 (52)
Unstable	76 (54)	58 (41)
Enforced	8 (6)	8 (6)
Other	0 (0)	1 (1)
Educational background, *n* (%)		
No qualifications	38 (27)	34 (24)
Basic school level qualifications	41 (29)	41 (29)
A'level or equivalent	10 (7)	12 (9)
Degree/professional qualification	51 (36)	53 (38)
In education prior to prison, *n* (%)		
Full-time/part-time education	10 (7)	4 (3)
Not in education	130 (93)	136 (97)
Pre-prison employment status, *n* (%)		
Full-time/part-time paid employment	28 (20)	40 (29)
Full-time/part-time self-employed	7 (6)	13 (9)
Other (e.g. voluntary, retired, carer)	1 (1)	2 (1)
Not working	104 (74)	85 (61)
Pre-prison income source, *n* (%)		
No source of income	22 (16)	11 (8)
Employment	30 (21)	40 (29)
Benefits	77 (55)	78 (56)
Other	11 (7)	11 (8)
Pre-prison income (£), *n* (%)^a^	(*N* = 138)	(*N* = 138)
Less than 13 500	114 (83)	107 (78)
13 501 or more	24 (17)	31 (22)
Marital status, *n* (%)		
Married	2 (1)	5 (4)
Cohabiting	15 (11)	14 (10)
Single	117 (84)	110 (79)
Divorced/separated	6 (4)	10 (7)
Widowed	0 (0)	1 (1)
At least one living parent: rare or no contact, *n* (%)	20/112 (18)	20/109 (18)
At least one sibling: rare or no contact, *n* (%)	28/126 (22)	35/131 (27)
Has physical health problems, *n* (%)	64 (46)	48 (34)
Has experienced trauma, *n* (%)		
Sexual	27 (19)	27 (19)
Relational	74 (53)	89 (64)
Other/no	39 (28)	24 (17)
Diagnosis of ADHD, *n* (%)	23/139 (17)	28/139 (20)
Diagnosis of autistic spectrum disorder, *n* (%)	1 (1)	5/139 (4)
Previous head injury, *n* (%)	113 (81)	110/139 (79)
Standard assessment of personality, mean (s.d.), *n*	4.3 (1.6), 138	4.5 (1.8), 139
Alcohol problem (self-report), *n* (%)	50/139 (36)	50 (36)
Drug problem (self-report), *n* (%)	69/139 (50)	60 (43)
History of self-harm, *n* (%)	63 (45)	73 (52)
Self-harm in past 3 months, *n* (%)	17 (12)	25 (18)
Self-harm with suicidal intent in last 3 months, *n* (%)	8/139 (6)	7 (5)

ADHD, attention-deficit hyperactivity disorder.

a. *n* for both groups for Pre-prison income (£) is 138.

There were between-group imbalances in participant characteristics at baseline for type of accommodation (the usual care group had more stable accommodation before prison), employment (the usual care group were more likely to be employed), previous trauma (the usual care group had experienced more relational trauma) and physical health problems (the usual care group had fewer problems). Hence, these characteristics were included as covariates in all analyses.

With regard to implementation of Engager, of the 140 participants allocated to the intervention 92% received at least one session in prison, with a mean of 5.7 sessions (s.d. = 3⋅9). Support on the day of release was carried out with 51% (*n* = 71). A total of 77% (*n* = 108) received at least one post-release community session, a mean of 12.9 (s.d. = 11.6) (see Supplementary Table 1). Less than half of participants (*n* = 62/129, 48%) received the minimum dose of the intervention seen as likely to be required to have an impact (two prison sessions and eight community sessions).

Usual mental healthcare receipt in both arms post release included contact with a range of services (see Supplementary Tables 2–3). After release the majority of individuals received usual mental healthcare in general practice (control group 65%, *n* = 61/94, intervention group 65%, (*n* = 64/98). Only 3% (*n* = 3/94) of controls and 6% (*n* = 6/98) receiving the intervention reported seeing psychiatrists, and 22% in the intervention group (*n* = 22/98) and 17% in the control group (*n* = 17/94) saw other mental health professionals.

The primary outcome was CORE-OM total score at 6-month follow-up. This was obtained for 184 participants (66%, 92 from each arm). There were no significant differences in mean CORE-OM total scores between the groups at the primary outcome time point for the ITT analysis (mean difference 1.1, 95% CI −1.1 to 3.2, *P* = 0.325) and also no significant difference at 12 months (see Table 2), or across time points in the repeated-measures analysis (see Supplementary Table 4).

**Table 2:** Clinical Outcomes in Routine Evaluation Outcome Measure (CORE-OM) at baseline and follow-up: intention-to-treat analyses using observed data only

	Time point											
	Baseline, mean (s.d.) *n*		1-month follow-up, mean (s.d.) *n*		3-month follow-up, mean (s.d.) *n*		6-month follow-up			12-month follow-up		
Outcome	Engager (*n* = 140)	Usual care (*n* = 140)	Engager (*n* = 88)	Usual care (*n* = 91)	Engager (*n* = 83)	Usual care (*n* = 96)	Engager, mean (s.d.) *n* (*n* = 92)	Usual care, mean (s.d.) *n* (*n* = 92)	Engager *v*. usual care, between-group mean difference (95% CI) *P*^a^	Engager, mean (s.d.) *n* (*n* = 67)	Usual care, mean (s.d.) *n* (*n* = 59)	Engager *v*. usual care, between-group mean difference (95% CI) *P*^a^
CORE-OM total	15.2 (6.0) 140	16.9 (6.2) 140	13.3 (8.6) 80	12.6 (8.4) 76	13.4 (7.2) 80	12.8 (7.8) 88	12.6 (6.9) 92	11.9 (7.7) 90	1.1 (−1.1 to 3.2) 0.325	11.2 (6.8) 66	12.0 (8.9) 58	−0.6 (−3.5 to 2.3) 0.700
CORE-OM wellbeing	16.8 (9.5) 140	19.6 (8.3) 139	162 (11.6) 78	14.6 (10.6) 76	15.9 (9.4) 79	14.1 (10.5) 87	14.5 (10.1) 91	14.0 (10) 89	0.9 (−2.0 to 3.9) 0.529	12.0 (8.7) 65	12.2 (11.3) 57	0.0 (−3.8 to 3.8) 0.994
CORE-OM symptoms	19.3 (8.1) 140	21.4 (8.6) 140	15.7 (10.8) 80	14.9 (10.3) 76	16.3 (9.8) 80	15.6 (9.7) 88	15.4 (8.5) 92	14.4 (9.5) 90	1.2 (−1.4 to 3.8) 0.352	14.1 (8.8) 66	14.0 (10.0) 58	0.6 (−3.0 to 4.2) 0.736
CORE-OM functioning	17.0 (6.9) 140	18.2 (6.7) 140	14.7 (9) 80	14.1 (9.2) 76	14.6 (7.4) 80	14.5 (8.5) 88	14.1 (7.5) 92	12.9 (8.2) 90	1.4 (−0.9 to 3.7) 0.241	12.6 (7.6) 66	13.4 (9.1) 58	−0.9 (−4.1 to 2.2) 0.559
CORE-OM risk	2.2 (3.6) 140	3.7 (5.3) 137	3.3 (5.4) 80	3.7 (5.8) 74	3.7 (4.8) 80	2.9 (5.3) 85	3.0 (4.8) 92	3.2 (5.3) 88	0.1 (−1.5 to 1.6) 0⋅910	2.7 (4.7) 66	4.9 (6.7) 58	−2.5 (−4.6 to −0.4) 0.020

1 Adjusted for site, baseline covariates (unstable accommodation, trauma (three categories), not working, physical health problem), baseline score.

**Fig. 2:**
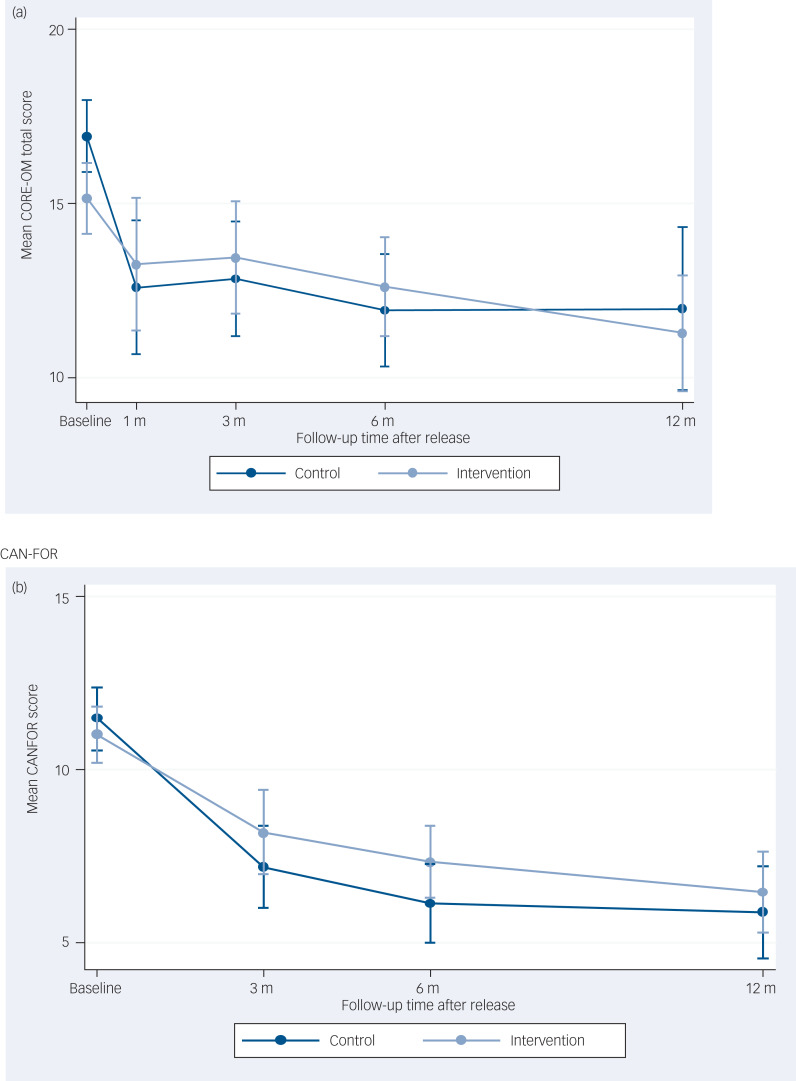
Graphs of outcomes over time in months for (a) Clinical Outcomes in Routine Evaluation Outcome Measure (CORE-OM) total and (b) Camberwell Assessment of Need – Forensic Version (CAN–FOR).

Fig. 2 depicts the change in CORE-OM and CAN–FOR over time.

The secondary outcomes are reported in Supplementary Tables 5–7. For one of the secondary outcomes, CORE-OM risk score, there was some evidence of a difference between groups at 12-month follow-up for the ITT analysis in favour of Engager (mean difference −2.5, 95% CI −4.6 to −0.4), *P* = 0.020. The per protocol and CACE analyses also produced evidence to indicate a difference between groups for CORE-OM risk score (Supplementary Table 8 and Supplementary Table 10).

The CORE-OM wellbeing scores were mostly found to be higher in the Engager group in the repeated-measures analysis (see Supplementary Table 4). There was a significant interaction effect between presence of an alcohol problem and intervention with regard to CORE-OM total score at 12-month follow-up, indicating that participants receiving the Engager intervention who also had an alcohol problem at baseline derived a greater treatment effect from the intervention compared with those who did not have an alcohol problem (Supplementary Table 11). However, because of the number of inferential analyses performed, including primary and secondary outcomes, types of analysis (ITT, per protocol and CACE), analyses at both 6- and 12-month follow-up, and subgroup analyses, we would expect to see some significant results owing to chance alone, and therefore these results should be interpreted in that light.

No reoffending data are presented here as data from the Police National Computer was not available. Serious adverse events were monitored and a total of 31 serious adverse events were reported (22 in the intervention group and 9 in the usual care group), 6 of which resulted in the death of the participant (4 in the intervention group and 2 in the control group). The trial steering committee concluded that there was no reason to believe that these serious adverse events were related to either the intervention or the trial.

## Discussion

Engager is the first RCT of a collaborative care intervention adapted for prison leavers with common mental health problems. There were 68% potential participants who agreed to be screened, of whom 73% met the criteria. Through researcher effort, follow-up rates of 66% at 6 months after release were achieved. There were some imbalances in characteristics at baseline, with participants in the Engager group having less stable accommodation, lower levels of employment and poorer physical health, whereas the usual care group had more trauma as well as higher levels of distress. The trial demonstrated no difference between Engager and usual care for the primary outcome.

We found that delivery of the intervention was inconsistent. Eleven (8%) participants received no prison sessions. There was significant variation in the number of meaningful community contacts received and 23% (*n* = 32) had no contact after release. Although the intervention was designed to be flexible according to need, this level of variability was more than intended. It may have been because of variation between practitioners, illness within and changes to the team, organisational constraints and the social adversity of the population. Arguably these levels of contact and engagement are typical for the context and population.

### Strengths and limitations

Strengths include high levels of recruitment and consent in this complex group, achieved by limiting exclusions and focusing on building trust. The follow-up rate of 66% at 6 months is high compared with most follow-up studies for prison leavers, except for those involving substance misuse or HIV treatment.^21,22^ Our ability to follow-up was based on extensive feasibility work and a substantial and proactive team of researchers.^10,11^ Our pre-trial work developing a theoretically informed,^16–18^ practically tested^11,18^ and then adapted intervention^18^ is a further strength. The explicitly flexible intervention designed to support personalised integrated care was developed with high fidelity to methods recommended by the Medical Research Council.^23^

There are limitations in both trial design and intervention theory and delivery. Although the follow-up may be considered impressive for offenders it is relatively low compared with collaborative care mental health studies with other populations.^12^ Masking was not possible for either participants or researchers, opening the trial to bias. However, reports from researchers indicated many participants in the control group thought the researchers were providing support, potentially weakening the ability to detect a difference. The baseline differences suggested that the Engager group was more socially excluded (homelessness in particular) but less distressed and traumatised. At follow-up a higher proportion of those in the intervention arm were followed up in prison, having re-offended or breached license conditions. This seems unlikely to be because of the intervention; however, in the absence of criminal justice data we were unable to test if it was because they were a more criminally active and experienced group. By contrast, the usual care group had more trauma, higher baseline mental health symptoms and a greater (but not statistically significant) improvement in mental health 3 months following release. Although the multiple baseline imbalances were adjusted for in the analysis, it is possible that further unmeasured differences had a small impact on outcomes. However, such imbalances are unlikely to have led to a clinically significant effect on the primary and secondary outcomes.

The outcomes used were potentially the greatest weakness of the trial. The person-centred intervention, designed to support whatever pathway an individual needed, created problems for measuring small but potentially critical changes for an individual. Although considerable effort was made to combine literature, piloting and stakeholder consensus in the selection of measures, it is possible that standard trial measures are not sensitive to the idiosyncratic responses to the intervention found in the process evaluation. Therefore, it is possible that any small steps towards recovery from lifelong adversity brought about by the intervention were not captured. The process evaluation, masked to individual outcome changes, described positive responses in a proportion of participants receiving a high-fidelity intervention.^24^ These responses were not associated at the individual level with improvements in the CORE-OM score, potentially because recovery pathways can involve acknowledgement or tolerance rather than resolution of distress.

Although considerable effort went into bringing together evidence from multiple sources to create the complex Engager intervention, there was little research to build on and it is possible that key aspects of intervention theory were suboptimal. Finally, despite the development of a substantive implementation delivery platform, implementation was inconsistent. This might have had an impact on the Engager programme achieving its full effect. The CACE and per protocol analyses would have partly addressed this issue but were carried out on the basis of ‘dose’ and not quality of intervention.^23^

### Interpretation and implications

The inconsistent delivery, baseline differences and likely problems with outcome measures complicate interpretation of the results. We have little research to compare Engager to as there have been no other RCTs of interventions for prison leavers with common mental health problems. It can be safely concluded that the Engager intervention as delivered at the time of the trial in 2016–2018 in a UK context was not effective at generating significant effects in the standard trial outcome measures that we used. Even if the limitations in study methods had been addressed, this conclusion holds firm. The potential weakness of the outcome measures means the intervention might have generated varied but individually valued benefits not detected with standard measures; whether improved delivery could have extended any such small benefits, and whether the cost of delivery would have made such hypothetical improvements worthwhile is not known.

The detailed process evaluation shows that more focus on ongoing training and supervisory support and a more favourable interorganisational context might have improved fidelity^25^ and provides further learning for UK policy of continuing mental healthcare on release from prison.^7^ It could be argued that Engager should have been targeted at those with more or less need (more scope to benefit, or easier to engage, respectively), however, the process evaluation indicated that those with better outcomes included those with higher or lower needs.^24^

It is possible that the intervention could be improved by strengthening, removing or adding components, perhaps making its simpler and easier to implement. There could also be a rationale for changing the delivery from the standalone Engager team used in the trial to a different and less costly model involving both the integration of poorly coordinated services (e.g. those addressing housing instability, substance misuse and unemployment) and adding in supervision for these existing workers who could be trained in Engager methods. The lack of similar interventions and trials means that it is also difficult to make strong recommendations for other counties.

Although resource intensive and requiring flexibility and resilience, research in the criminal justice system was generally welcomed. Further research, potentially using different methods or testing ‘integration’ rather than the separate Engager team, is needed to determine whether mental health supported ‘through-the-gate’ and post-release interventions can be beneficial and justified. It will also be important to more formally test mentalisation-based treatment or other promising psychological therapies in this population, although substantial adaptation would be required to provide a flexible mode of delivery.

In conclusion, this was the first RCT of a complex ‘through-the-gate’ intervention for prison leavers with common mental health problems; it was successfully completed within challenging health and criminal justice systems. No improvements in traditional mental health-specific- and multidomain-outcome measures were seen. However, given this is an underresearched high-need population further formal research testing interventions and pragmatic mixed-methods evaluation and implementation research would be helpful in order to gain insights from stressed and unpredictably dynamic justice health and social care systems.

## Data Availability

The study protocol has been published and the statistical analysis plan is part of supplementary material. Anonymised data may be made available by request to the corresponding author. Changes from the original funding proposal include, following pilot work, a funded extension for a trial based on health outcomes (rather than exploratory trial as originally funded) as described in the published protocol, trial registry and statistical analysis plan. An application for trial registration was made in December 2015 in the normal way through the NIHR portfolio registration system (before recruitment) but due to administrative delays the ISRCTN registration date was 4/02/2016 while recruitment started 14/1/2016. The discrepancies from the published protocol included provision of ‘top-up’ training for new and existing practitioners during the trial and provision of ‘meta-supervision’ to support Engager team leader supervisors to overcome ongoing operational problems in order to optimise (but not change) intervention delivery. In addition reoffending data, of the secondary outcomes, was not made available by the Police National Database as planned and is still not available.

## Supplementary material

For supplementary material accompanying this paper visit https://doi.org/10.1192/bjp.2022.93. click here to view supplementary material
