# Supplementary material for: Evaluation of a complex intervention for prisoners with common mental health problems, near to and after release: the Engager randomised controlled trial
Source: Br J Psychiatry. 2023 Jan;222(1):18–26. doi: 10.1192/bjp.2022.93 (PMC10895504; doi:10.1192/bjp.2022.93)
Supplement: Supplementary file 1 [file S0007125022000939sup001.docx]

**Supplementary Material**

**Contents Page**

[1.1 Consort Checklist 2](#_Toc101988819)

[1.2 Eligibility Assessment 4](#_Toc101988820)

[1.3 Usual care description 5](#_Toc101988821)

[1.4 Description of outcome measures 6](#_Toc101988822)

[1.5 Statistical Analysis Plan 8](#_Toc101988823)

[1.6 Service Use Data for intervention and usual care 39](#_Toc101988824)

[1.7 Intention to treat analyses 41](#_Toc101988825)

[1.8 Per protocol analyses 45](#_Toc101988826)

[1.9 CACE and interactional analyses 48](#_Toc101988827)

1.1 Consort Checklist


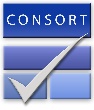
CONSORT 2010 checklist of information to include when reporting a randomised trial*

| Section/Topic | Item No | Checklist item | Reported on page No |
| --- | --- | --- | --- |
| Title and abstract | | | |
|  | 1a | Identification as a randomised trial in the title | 1 |
|  | 1b | Structured summary of trial design, methods, results, and conclusions (for specific guidance see CONSORT for abstracts) | 2 |
| Introduction | | | |
| Background and objectives | 2a | Scientific background and explanation of rationale | 2-3 |
|  | 2b | Specific objectives or hypotheses | 3 |
| Methods | | | |
| Trial design | 3a | Description of trial design (such as parallel, factorial) including allocation ratio | 3 |
|  | 3b | Important changes to methods after trial commencement (such as eligibility criteria), with reasons | Not applicable |
| Participants | 4a | Eligibility criteria for participants | 3 |
|  | 4b | Settings and locations where the data were collected | 3 |
| Interventions | 5 | The interventions for each group with sufficient details to allow replication, including how and when they were actually administered | 4 |
| Outcomes | 6a | Completely defined pre-specified primary and secondary outcome measures, including how and when they were assessed | 4 |
|  | 6b | Any changes to trial outcomes after the trial commenced, with reasons | Not applicable |
| Sample size | 7a | How sample size was determined | 4 |
|  | 7b | When applicable, explanation of any interim analyses and stopping guidelines | Not applicable |
| Randomisation: |  |  |  |
| Sequence generation | 8a | Method used to generate the random allocation sequence | 4 |
|  | 8b | Type of randomisation; details of any restriction (such as blocking and block size) | 4 |
| Allocation concealment mechanism | 9 | Mechanism used to implement the random allocation sequence (such as sequentially numbered containers), describing any steps taken to conceal the sequence until interventions were assigned | 4 |
| Implementation | 10 | Who generated the random allocation sequence, who enrolled participants, and who assigned participants to interventions | 3 |
| Blinding | 11a | If done, who was blinded after assignment to interventions (for example, participants, care providers, those assessing outcomes) and how | 3 |
|  | 11b | If relevant, description of the similarity of interventions | Not applicable |
| Statistical methods | 12a | Statistical methods used to compare groups for primary and secondary outcomes | 4-5 |
|  | 12b | Methods for additional analyses, such as subgroup analyses and adjusted analyses | 5 |
| Results | | | |
| Participant flow (a diagram is strongly recommended) | 13a | For each group, the numbers of participants who were randomly assigned, received intended treatment, and were analysed for the primary outcome | 5-6 |
|  | 13b | For each group, losses and exclusions after randomisation, together with reasons | 5-6 |
| Recruitment | 14a | Dates defining the periods of recruitment and follow-up | 5 |
|  | 14b | Why the trial ended or was stopped | Not applicable |
| Baseline data | 15 | A table showing baseline demographic and clinical characteristics for each group | 7 |
| Numbers analysed | 16 | For each group, number of participants (denominator) included in each analysis and whether the analysis was by original assigned groups | 7 |
| Outcomes and estimation | 17a | For each primary and secondary outcome, results for each group, and the estimated effect size and its precision (such as 95% confidence interval) | 7.8 and supplementary p 43 |
|  | 17b | For binary outcomes, presentation of both absolute and relative effect sizes is recommended | Not applicable |
| Ancillary analyses | 18 | Results of any other analyses performed, including subgroup analyses and adjusted analyses, distinguishing pre-specified from exploratory | 8 |
| Harms | 19 | All important harms or unintended effects in each group (for specific guidance see CONSORT for harms) | 8 |
| Discussion | | | |
| Limitations | 20 | Trial limitations, addressing sources of potential bias, imprecision, and, if relevant, multiplicity of analyses | 11 |
| Generalisability | 21 | Generalisability (external validity, applicability) of the trial findings | 12 |
| Interpretation | 22 | Interpretation consistent with results, balancing benefits and harms, and considering other relevant evidence | 12 |
| Other information | | |  |
| Registration | 23 | Registration number and name of trial registry | 3 |
| Protocol | 24 | Where the full trial protocol can be accessed, if available | 3 |
| Funding | 25 | Sources of funding and other support (such as supply of drugs), role of funders | 14 |

*We strongly recommend reading this statement in conjunction with the CONSORT 2010 Explanation and Elaboration for important clarifications on all the items. If relevant, we also recommend reading CONSORT extensions for cluster randomised trials, non-inferiority and equivalence trials, non-pharmacological treatments, herbal interventions, and pragmatic trials. Additional extensions are forthcoming: for those and for up to date references relevant to this checklist, see [www.consort-statement.org](http://www.consort-statement.org).

1.2 Eligibility Assessment

| **Criteria** | **How assessed** |
| --- | --- |
| Men serving a prison sentence of two years or less | All prisons were male establishment. Using the Prison National Offender Management Information System p-NOMIS the researchers could search for all prisoners in the prison by sentence length. |
| Between four and twenty weeks remaining until anticipated release date | Using p-NOMIS the researchers could search from prisoners with released dates within a date range. |
| Being released to the geographical area of the study | Using p-NOMIS the researchers could see the prisoners address on reception into prison. The researchers had a list of postcodes of the geographical area of release. |
| Willing to engage with treatment services and research procedures | Participants consented to take part. |
| Identified as having, or likely to have following release, common mental health problems | All individuals providing written informed consent completed a short screening interview with the researchers to identify those currently experiencing common mental health problems or who had experienced common mental health problems in the previous 2 years that impacted on their day-to-day functioning and were likely to experience similar problems on release. The screening interview comprises the Patient Health Questionnaire-9 (PHQ-9), the Generalised Anxiety Disorder-7 (GAD-7), the Primary Care PTSD Screen (PC-PTSD) and a bespoke Historical Common Mental Health Problem screen.  Individuals were considered suitable for inclusion in the study if the screening interview indicated that they:  have a current common mental health problem as indicated by a score of 10 or more on the PHQ-9 or GAD-7, or 3 or more on the PC-PTSD; or  have experienced a common mental health problem during the past 2 years, which prevented them from functioning normally in everyday tasks and which is likely to be a problem for them again following their release. |
| Men on remand | Using p-NOMIS the researchers could see the status of all prisoners and exclude those on remand. If men on remand became sentenced during the study they then became eligible to take part. The rational for excluding those on remand, even though we know mental health needs and risk of suicide/self-harm is greater, but the unpredictability of release meant it would limit the delivery of the Engager intervention. |
| Serious and enduring mental disorder and/or on the caseload of the prison in-reach team | The researchers accessed the NHS trusts clinical records system (SystmOne) to see if participants had been previously diagnosed with a serious and enduing mental disorder (e.g. psychosis) and/or they were currently on the caseload of the prison in-reach team. |
| Primary personality disorder who were on the caseload of the Offender Personality Disorder Pathway service | The researchers accessed the NHS trusts clinical records system to see if participants were on the caseload of the Offender Personality Disorder Pathway service. |
| Active suicidal intent requiring management under the safer custody process and where the healthcare team felt participation in the study would be detrimental | The researchers asked the safer custody department within the prison to indicate any people with active suicidal intent and asked healthcare staff if participation was suitable. |
| Serious risk of harm to the researchers or intervention practitioners | The researchers accessed p-NOMIS which contained information on those prisoners who posed a risk. |
| Unable to provide informed consent | Researcher were trained to assess capacity to consent. This included assessing if the prisoner had the ability to understand the information relevant to the decision, retain the information, use or weigh the information as part of the process for decision making and communicate the decision to the researcher. The researchers also sort advice from the healthcare team. |

1.3 Usual care description

Individuals in the control group received usual care. While still in prison, they were able to access primary care, secondary mental health and substance misuse services as would usually be the case. They also received support from criminal justice and any other third-sector organisations in the standard way.

***Northwest***

**Prison**

This is a Category B local prison, meaning that it takes prisoners directly from court in the local area (sentenced or on remand). The prison is run by HMPPS and has a capacity of 1173. At the time of the study, a local NHS Trust provided all health provision (primary, secondary and substance misuse). A local college provided education within the prison with courses offered based around the key areas of functional skills, vocational training, information technology and employability. A national third-sector organisation provided accommodation support and the National Probation Service or Community Rehabilitation Company provided offender management (depending on the offence and risk of the individual). In addition, people could access listeners (prisoner volunteers trained to listen to fellow inmates who are in distress, similar to Samaritan volunteers), chaplaincy, gym and work.

**Community**

In the community all men were either supervised for a period of time by the NPS or CRC. A probation case manager was appointed to work with them while in prison and develop a resettlement plan. This could cover issues such as accommodation, debt and educational advice. They would also monitor adherence to any licence conditions.

Released men had access to numerous community services catering for a range of needs. These included: Shelter and the Whitechapel Centre (accommodation and homeless services); National Careers Service, Job Centre, Sefton CVS, Achieve Northwest, AIMS Local Solutions and Working Links (employment, benefits, volunteering and welfare services); Addaction, Lifeline, Brook Place, Narcotics Anonymous and Alcoholics Anonymous (substance misuse services); and SSAFA, Royal British Legion, Salvation Army and Speke House (Veterans and welfare services). In addition, participants had access to community mental health and counselling services. Schemes were also available where released men could give or receive mentorship in various work or life-skill domains.

***Southwest***

**Prison**

This is a Category B local prison, meaning that it takes prisoners directly from court in the local area (sentenced or on remand). The prison is run by HMPPS and has a capacity of 560. At the time of the study, a consortia of providers operated (primary, secondary and substance misuse). A national third-sector organisation provided accommodation support and the National Probation Service or Community Rehabilitation Company provided offender management (depending on the offence and risk of the individual). In addition, people could access listeners (prisoner volunteers trained to listen to fellow inmates who are in distress, similar to Samaritan volunteers), chaplaincy, gym and work.

**Community**

In the community all men were either supervised for a period of time by the NPS or CRC. A probation case manager was appointed to work with them while in prison and develop a resettlement plan. This could cover issues such as accommodation, debt and educational advice. They would also monitor adherence to any licence conditions.

Released men had access to numerous community services catering for a range of needs across the two counties in the study release area. These included a variety of housing, substance misuse, finance support and Royal British Legion.

1.4 Description of outcome measures

| **Measure** | **Abbreviation** | **Concept to be measured** | **Description** | **Details of adaptions** | **Scoring (range, directionality MCID)** | **Time points outcome collected** |
| --- | --- | --- | --- | --- | --- | --- |
| Clinical Outcomes in Routine Evaluation Outcome Measure^1^ | CORE-OM | Psychological distress | A 34-item scale comprising four domains: subjective wellbeing; depression and anxiety related problems and symptoms; general, social and relationship functioning; and risk of harm to self or others | None | Five point Likert scale ranging from 'not at all' to 'most or all of the time'.  The mean across the items, i.e. between 0 and 4 is calculated and multiplied by ten, giving scores between 0 and 40. A higher score indicates higher distress.  MCID = 5 | Baseline; Pre-release; 1, 3, 6, & 12 month |
| Camberwell Assessment of Need – Forensic Version^2^ | CANFOR | Met and unmet need | A needs assessment across 25 areas of life and health. | Areas of need were removed if not relevant to a common mental health population or where there was no/low needs identified in the pilot trial. Items removed were: psychotic symptoms, information on condition and treatment, treatment (i.e. does the person agree with the treatment prescribed to them), and sexual expression.  The response format was shortened to better fit the service setting and for use in the time-limited prison regime. Responses were specified and participants were asked whether, in a particular domain, there is no need, moderate need or high need, whether they are receiving support for the need and whether the support is reducing that need. This was then amalgamated into a single overall response . | A need is deﬁned as being present when the interviewee indicates that there have been difﬁculties in a  particular area over the last month. If a need is deemed  present, the domain is then scored as either met or  unmet. A met need is deﬁned where a difﬁculty has  been identiﬁed for which an appropriate intervention  is currently being received. An unmet need is deﬁned  where a difﬁculty has been identiﬁed for which no  interventions are currently being received, or that any interventions or  support being received are not helping. If a need is not  considered to be present it is scored as no need or,  in certain instances, not applicable. The total need  score is deﬁned as the sum of the number of met needs  and unmet needs | Baseline; 3, 6 & 12 month |
| Treatment Outcomes Profile^3^ | TOP | Drug and alcohol use | Assessment to measure substance use and substance use treatment. | Yes | Uses a time-line follow back to record days used alcohol, illicit opiates, cocaine, non-prescribed drugs, amphetamine and cannabis over the last 28 days. Estimates of total quantity used on a typical day were recorded. | Baseline; 3, 6, & 12 month |
| Leeds Dependence Questionnaire^4^ | LDQ | Drug and alcohol dependence | A 10-item, self-completion questionnaire designed to measure dependence upon a variety of substances. | None | Items are scored 0-1-2-3 to create a total score. | Baseline; 6 & 12 months |
| EuroQol–5 Dimensions–5  Levels^5^ | EQ-5D-5L | Health-related quality of life | A five-item questionnaire measuring: mobility, self-care, usual activities, pain/discomfort and anxiety/depression. | None | Each dimension has 5 levels: no problems, slight problems, moderate problems, severe problems and extreme problems. | Baseline, 3, 6, and 12 months |
| ICEpop CAPability^6^ | ICE-CAP-A | Well-being related quality of life | A five-item questionnaire measuring: attachment, stability, achievement, enjoyment, autonomy. | None | Each capability item has four levels of responses. | Baseline, 3, 6, and 12 months |
| Intermediate Outcomes Measurement Instrument^7^ | IOMI | Psychological constructs related to desistance |  | No changes made to items, but decision made to report as one total score rather than subscores to reduce numbers of secondary outcomes. |  | Baseline; 6 & 12 months |
| Brief INSPIRE^8^ | n/a | Subjective experience of care received | Five item questionnaire | Yes. Adapted to rate services rather than single worker. | One point per item. | Pre-release, 3, 6 and 12 months |
| Client Service Receipt Inventory^9^ | CSRI | Service use across health, criminal justice, social care and third sector organisations and helpfulness of services |  | Yes. A helpfulness score was added for each contact. |  | Pre-release, 3, 6 and 12 months |
| Police National Computer | PNC | Reoffending |  | Unable to obtain permission within programme period | N/A | N/A |

References:

1. Evans C, Mellor-Clark J, Margison F et al. CORE: Clinical outcomes in routine evaluation. *Journal of Mental Health*. 2000;**9**:247
2. Thomas S, Harty MA, Parrott J et al. CANFOR: Camberwell assessment of need-forensic version. London: Gaskell, 2003.
3. Marsden J, Farrell M, Bradbury C et al. Development of the treatment outcomes profile. *Addition*. 2008;**103**:1450–60.
4. Raistrick D, Bradshaw J, Tober G et al. Development of the leeds dependence questionnaire (ldq): A questionnaire to measure alcohol and opiate dependence in the context of a treatment evaluation package. *Addiction*. 1994;**89**:563–72.
5. Herdman M, Gudex C, Lloyd A et al. Development and preliminary testing of the new five-level version of EQ-5D (EQ-5D-5L). *Quality of Life Research*. 2011;**20**:1727–36.
6. Al-Janabi H, Flynn T, Coast J. [Development of a self-report measure of capability wellbeing for adults: the ICECAP-A](http://link.springer.com/article/10.1007/s11136-011-9927-2). *Quality of Life Research*. 2012;**21**:167-176.
7. Maguire M, Disley E, Liddle M et al . *Developing a toolkit to measure intermediate outcomes to reduce reoffending*. London: Ministry of Justice Analytical Series, 2017.
8. Williams J, Leamy M, Bird V, Le Boutillier C, Norton S, Pesola F, Slade M (2015) Development and evaluation of the INSPIRE measure of staff support for personal recovery. *Social Psychiatry and Psychiatric Epidemiology*, **50**, 777-786.
9. Beecham J, Knapp M. Costing psychiatric interventions. In: Thornicroft G, ed. Measuring Mental Health Needs. London: Gaskel, 2001.

1.5 Statistical Analysis Plan

| 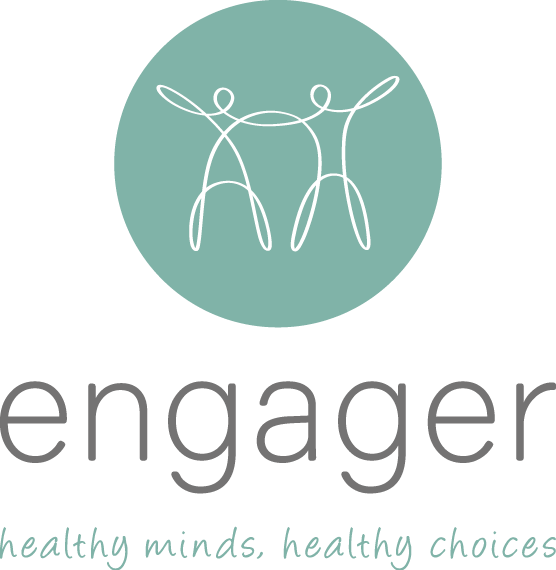 |
| --- |
|  |
| Evaluation of a complex intervention (Engager) for prisoners with common mental health problems, near to and after release |

Statistical Analysis Plan

Version 1.1.6

20 February 2019

1. Trial data

| Trial full title | Evaluation of a complex intervention (Engager) for prisoners with common mental health problems, near to and after release |
| --- | --- |
| Trial registration number | ISRCTN11707331 |
| Trial chief investigator | Prof Richard Byng |
| Trial manager | Dr Tim Kirkpatrick |
| Trial statistician | Prof Rod Taylor |
| SAP author | Dr Fiona Warren |
| CTU involvement (name of CTU and role, e.g. data management, randomisation) | PenCTU: randomisation, data management |

2. Abbreviations and definitions

All abbreviations used in this SAP are defined in Table 1.

**Table 1 Abbreviations**

| **Abbreviation** | **Definition** |
| --- | --- |
| AE | Adverse Event |
| CAN-FOR | Camberwell Assessment of Need – Forensic Version |
| CBT | Cognitive Behavioural Therapy |
| CI | Chief Investigator |
| CJS | Criminal Justice System |
| CMHP | Common Mental Health Problem |
| ConSORT | Consolidated Standards of Reporting Trials |
| CORE-10 | Clinical Outcomes in Routine Evaluation – 10 item version |
| CORE-OM | Clinical Outcomes in Routine Evaluation – Outcome Measure |
| CRF | Case Report Form |
| CRN | Clinical Research Network |
| CSRI | Client Service Receipt Inventory |
| CTU | Clinical Trials Unit |
| DMC | Data Monitoring Committee |
| DPT | Devon Partnership NHS Trust |
| EQ-5D-5L | European Quality of Life-5 Dimensions- 5 Levels |
| FPE | Formative Process Evaluation |
| GCP | Good Clinical Practice |
| GP | General Practitioner |
| IAPT | Improving Access to Psychological Therapies |
| ICE-CAP-A | ICEpop CAPability measure for Adults |
| ICH GCP | International Conference on Harmonisation of Good Clinical Practice |
| IOMI | Intermediate Outcomes Measurement Instrument |
| ITT | Intention to Treat |
| LDQ | Leeds Dependence Questionnaire |
| MCID | Minimum Clinically Important Difference |
| NIHR | National Institute of Health Research |
| NOMS | National Offender Management Service |
| NRES | National Research Ethics Service |
| PenCTU | Peninsula Clinical Trials Unit |
| PNC | Police National Computer |
| PNOMIS | Prison National Offender Management Information System |
| PPI | Patient and Public Involvement |
| PSC | Programme Steering Committee |
| PIS | Patient Information Sheet |
| PTSD | Post Traumatic Stress Disorder |
| QALY | Quality Adjusted Life Year |
| R&D | Research and development |
| RCT | Randomised controlled trial |
| REC | Research Ethics Committee |
| SAE | Serious Adverse Event |
| SAP | Statistical Analysis Plan |
| SD | Standard Deviation |
| SOP | Standard Operating Procedure |
| SPCR | Surveying Prisoner Crime Reduction |
| SUSAR | Suspected Unexpected Serious Adverse Reaction |
| TOG | Trial Oversight Group |
| TOP | Treatment Outcomes Profile |
| TSC | Trial Steering Committee |
| UKCRC | United Kingdom Clinical Research Collaboration |

3. Statistical guidelines

Analyses are to be conducted in accordance with ICH-9 statistical guidelines for clinical trials and results are to be reported in accordance with the CONSORT checklist for trials [1, 2].

4. Trial background

This trial evaluates the Engager intervention for offenders in prison who are due to be released between 4 and 20 weeks from recruitment. This is a fully powered trial and is funded by the NIHR as part of a Programme Grant of Applied Research (RP-PG-1210-12011). The Engager trial is fully described in the protocol; this SAP relates to Protocol version 5 (19/04/2017). The Engager intervention is aimed at prisoners who have or are at risk of common mental health issues on release from prison, which could adversely affect physical health and other social outcomes such as employment or risk of reoffending. The Engager intervention is intended to have an effect on physical and mental health as well as outcomes such as social and employment outcomes, and reoffending.

5. Trial information

5.1 Interventions

This is a 2-arm trial, the control arm receives usual care only (care in prison and GP care plus other care from other agencies on release). The intervention arm will receive usual care plus the Engager intervention which includes support from a health care practitioner who will provide mental health support and also signposting to other sources of support. The Engager intervention is a manualised intervention delivered by mental health practitioners with appropriate supervision.

5.2 Phase of trial

This trial is a fully powered superiority trial. It is intended to recruit 280 patients across two sites (Devon and Manchester).

5.3 Randomisation level

The trial is randomised individually to either usual care or Engager plus usual care, in a 1:1 ratio. Randomisation will be stratified by site (Devon, Manchester). Randomisation is to be performed using a secure website created and managed by the PenCTU data management team. Randomisation will be performed by the recruiting researcher following completion of baseline screening and data collection.

5.4 Study design

The Engager trial is a 2-arm parallel individually randomised trial, being conducted at two sites. Due to the nature of the intervention it is not possible to blind participants to their intervention allocation. It is felt that researchers benefit from maintaining a relationship with participants and therefore it is not feasible to blind the researchers to the participant’s allocation. This raises issues surrounding bias in data collection; strategies to mitigate bias are described in the protocol. The data analysis will be performed by a statistician who is blinded to treatment allocation.

Participants will be randomised following screening to determine eligibility, provision of informed consent, and collection of baseline data. The Engager intervention will be provided prior to release (16 to 4 weeks) and after release for 8 weeks (with an optional continuation of the intervention at a lower intensity for a further 8 to 12 weeks).

Data collection will take place at baseline, and then at 1, 3, 6 and 12 months post release. The primary outcome timepoint is 6 months post release.

5.5 Purpose of the analyses

The purposes of the statistical analyses are as follows:

1. to provide descriptive baseline data by trial arm;
2. to report attrition at all follow-up timepoints;
3. to report individual outcome missingness at all follow-up timepoints;
4. to evaluate effectiveness of the intervention relative to control using inferential analyses;
5. to perform additional analyses to investigate potential moderators of the intervention effect; and
6. to provide descriptive data on serious adverse events.

Details of the quantitative mediation analyses will be described in a separate mediation analysis plan.

5.6 Sample size calculation

The trial is powered to detect an MCID of 3.5 on the CORE-OM scale (primary outcome), with an assumed SD of 7.5, with 90% power and a 2-sided alpha of 5%. The power calculation assumes 30% attrition at 6-month follow-up (the primary data collection time point).

5.7 Study populations

The study population is defined as male prisoners within 4 to 20 weeks of release with common mental health problems. Full inclusion/exclusion criteria are set out in the protocol V5. The full analysis population is defined on an intention-to-treat (ITT) basis, and will consist of all participants according to their randomised allocation. A per protocol analysis will be performed for the primary and secondary outcomes, including only those participants in the intervention arm who received a specified level of the intervention. For the intervention arm, participants will be considered to have received `per protocol’ treatment if they received at least two contacts prior to initial release and at least eight contacts following initial release (irrespective of location of contacts). All contact types will be included (i.e. face-to-face and telephone). Contacts on the day of initial release will be counted as post-release contacts. All patients in the control (usual care) arm are considered to be treated per protocol. All participants in the control arm will be considered to have received usual care (irrespective of variations to usual care actually received). Serious adverse events will be reported descriptively for all participants. Participants found to be ineligible after randomisation will be excluded from all analyses, but will be reported, with reasons for ineligibility.

6. Study objectives and endpoints

6.1 Study objectives

The objectives of the Engager trial (taken from protocol V5) are set out below.

1. To compare levels of psychological global distress between intervention and control

participants

1. To compare the number of subjective met and unmet needs in relation to

accommodation, education, work/money/benefits, family/friends/company/intimacy,

physical and mental health, safety to self and self-care, safety to others, and leisure

activities between intervention and control participants.

1. To compare substance use and subjective view of addiction between intervention

and control participants.

1. To compare levels of recidivism between intervention and control participants.
2. To compare generic health related quality of life between intervention and control

participants.

1. To compare the cost of health, social care, and criminal justice service utilisation

between intervention and control participants.

1. To compare subjective experience of care received between intervention and control

participants.

1. To compare perceived helpfulness of services engaged with between intervention

and control participants.

9. To complete a parallel process evaluation:

(i) to determine the degree to which the core mechanisms of the intervention were delivered

(ii) to evaluate the extent to which the core mechanism of the intervention produced

the intended outcomes

(iii) to identify aspects of the intervention and delivery that could be improved

(iii) to explore unintended consequences of the intervention

This SAP contributes to objectives 1, 2, 3, 4, 5, 7 and 8. Objective 9 will be in part evaluated by a quantitative mediation analysis.

6.2 Endpoints

Primary and secondary endpoints are set out in Table 2.

**Table 2 Endpoints**

| **Endpoint** | **Status** | **Data type** | **Source** | **Timepoints collected** |
| --- | --- | --- | --- | --- |
| CORE-OM | Primary | Continuous | Self-report; questionnaire | Baseline, 1, 3, 6, and 12 months |
| CORE-OM Wellbeing | Secondary | Continuous | Self-report questionnaire | Baseline, 1, 3, 6, and 12 months |
| CORE-OM Symptoms | Secondary | Continuous | Self-report questionnaire | Baseline, 1, 3, 6, and 12 months |
| CORE-OM Functioning | Secondary | Continuous | Self-report questionnaire | Baseline, 1, 3, 6, and 12 months |
| CORE-OM Risk | Secondary | Continuous | Self-report questionnaire | Baseline, 1, 3, 6, and 12 months |
| CORE-10 | Secondary | Continuous | Self-report questionnaire | Baseline, 1, 3, 6, and 12 months |
| CAN-FOR | Secondary | Continuous | Self-report questionnaire | Baseline, 3, 6, and 12 months |
| Social outcomes  Housing: Yes | Secondary | Binary | Self-report questionnaire | Baseline, 6, and 12 months |
| TOP | Secondary | Continuous (total number days abstinent (for relevant substance) over past 28 days) | Self-report questionnaire | Baseline, 3, 6, and 12 months |
| LDQ | Secondary | Continuous | Self-report questionnaire | Baseline, 6, and 12 months |
| EQ-5D-5L | Secondary | Continuous | Self-report questionnaire | Baseline, 3, 6, and 12 months |
| ICECAP-A | Secondary | Continuous | Self-report questionnaire | Baseline, 3, 6, and 12 months |
| IOMI | Secondary | Continuous | Self-report questionnaire | Baseline, 6, and 12 months |
| Brief INSPIRE | Secondary | Continuous | Self-report questionnaire | Pre-release, 3, 6 and 12 months |
| Helpfulness of services (CSRI) | Secondary | Continuous | Self-report questionnaire | Pre-release, 3, 6 and 12 months |
| Re-offending | Secondary | Binary | Police database | 12 months |

CAN-FOR: Camberwell Assessment of Need – Forensic Version. EQ-5D-5L: European Quality of Life 5 Dimensions – 5 Levels. ICECAP-A: ICEpop CAPability measure for Adults. IOMI: Intermediate Outcomes Measurement Instrument. LDQ: Leeds Dependent Questionnaire. TOP: Treatment Outcomes Profile.

We will also report location of participant at follow-up (in community or in prison) at 3- , 6- and 12-month follow-up, as reported by the researcher, as an item of interest, although not a formal outcome of the trial.

6.3 Derived variables

No derived variables are planned to be included in the analyses.

7 General analysis considerations

7.1 Timing of analyses

The analyses of data collected up to and including the primary analysis time point at 6 months will be performed when 6-month data becomes available. At this point the statistician and other triallists will be blinded to intervention allocation. Following discussion of blinded results, the arm intervention will be revealed. Results of data collected at 12-month follow-up will be analysed when data are available and presented with group allocation revealed. No interim data analyses are planned.

7.2 Types of analyses

The primary inferential analysis for primary and secondary outcomes will be the intention to treat analysis (i.e. according to initial random allocation) using complete case data for the eligible population at 6 months’ follow-up. All primary analyses will be performed using regression modelling appropriate to the data, i.e. linear regression modelling for continuous outcomes, and logistic regression for binary outcomes.

Secondary analyses for all primary and secondary outcomes with data available at any of the four follow-up time points will be undertaken as mixed effects repeated measures analyses. A per protocol analysis (excluding all participants in the treatment group who do not adhere to the intervention, as defined above) and a Complier Average Causal Effect (CACE) analysis of 6-month follow-up data will also be performed for the primary and secondary outcomes.

All analyses performed at 6-month follow-up will be repeated at 12-month follow-up.

A sensitivity analysis will be performed excluding those patients who were interviewed outside the follow-up `window’ for the primary outcome ITT analysis only, at 6- and 12-month follow-up.

7.3 Covariates and subgroups

All analyses will adjust for baseline values of continuous covariates and study site. Baseline characteristics will be evaluated for imbalance, and should any imbalances be observed for covariates that may be associated with outcomes, these characteristics will be included in the analyses if thought to be predictive of outcome.

For the CORE-OM at 6- and 12-month follow-up, interactions with intervention (i.e. moderation effects) will be sought with regard to study site, personality disorder (SAPAS), previous trauma, pre-prison accommodation and alcohol/substance abuse. A series of models will be performed, including one interaction term, between intervention and the covariate of interest, as well as other predictor variables included in the primary models, i.e. site, baseline CORE-OM and unbalanced covariates. Interaction effects will be reported using the coefficient for the interaction term with 95% confidence interval (CI) and global p-value for categorical variables with more than two categories. As subgroup analyses are not formally powered, such an analysis will be regarded as exploratory and hypothesis generating. For patients who received the Engager intervention only, we will consider investigating whether the degree of engagement with the intervention, based on the number of sessions completed, is associated with CORE-OM score.

7.4 Presentation of inferential analyses

Results of all inferential analyses will be reported as between group differences with 95% CIs; global *p-*values will also be provided with regard to categorical explanatory variables. The threshold for determining significant effects will be *p*< 0.05. No adjustment of *p*-values will be made to account for multiple testing, although the implications of multiple testing will be considered when evaluating the results of the analyses. Analysis of the primary outcome will be performed prior to all other analyses.

7.5 Missing data

The primary analysis for the primary outcome (CORE-OM at 6-month follow-up) will be based on complete case data. The sample size calculation (140 participants in each arm) was calculated with an inflation of 30% to account for attrition. Hence, 97 participants per arm will be sufficient to detect the MCID using MLHFQ score with 90% power. Loss to follow-up is not monotonic, participants may provide follow-up data for any combination of follow-up timepoints.

For both primary and secondary outcomes, the level of missingness will be evaluated, and the use of imputed data considered. The decision on imputation will be made considering the amount and nature of missing data, for example, the pattern of attrition across groups and the baseline characteristics of non-completers compared with completers. If imputation is performed, details of the imputation methods will be provided. If the amount of missing data is very small, it may be deemed more appropriate not to implement imputation.

Due to a high proportion of missing data (55% CORE-OM data missing at 12 months), data was imputed using chained equations, assuming that data was missing at random. The number of imputed datasets was based on the percentage of missing data at 12 months on the CORE-OM (i.e. 55 imputed datasets). Predictive mean matching was used for imputation of continuous outcomes.

7.6 Serious Adverse events

Data on serious adverse events will be set out descriptively by trial arm.

7.7 Reporting conventions

*P*-values ≥ 0.001 to will be reported to 3 decimal places, *p-*values<0.001 will be reported as “<0.001”. Quantiles, such as the median, or minimum and maximum, will be reported to the same number of decimal places as the original data. The mean, standard deviation and other statistics will be reported to one decimal place greater than the original data. For interaction (moderation) analyses, global *p*-values will be reported for interaction effects between intervention status and covariate, with marginal means and associated plots if beneficial.

7.8 Mediation analyses

Quantitative mediational analyses will be described in the process evaluation analysis plan.

7.9 Execution of analyses

The initial ITT analyses (after 6-month follow-up) will be performed by a statistician who is blinded to intervention allocation. Subsequent analyses will be performed after the treatment allocation has been revealed to the statistician. All analyses will be performed using Stata v.14.

7.10 Post hoc analyses on the primary outcome

Following presentation of the 6-month follow-up results, some additional analyses were considered to be potentially informative to understand the reasons for the results observed. Location of the participant at follow-up (in prison or in the community) was thought to potentially have an effect on the primary outcome of CORE-OM (at the group level, for example by including in the sample participants located in prison at follow-up who would not have been followed up in the community, and at the individual level due to the prison environment influencing CORE-OM responses). In addition, it was observed that a greater proportion of participants at 6-month follow-up were located in prison in the intervention group (36%) compared with the control group (20%). In view of this, we will add the following post hoc analyses: (1) report descriptively the baseline demographic characteristics of participants located in prison and in the community at 6-month follow-up. (2) We will then perform a regression analysis on the CORE-OM data at 6-month follow-up to investigate whether location at 6-month follow-up (prison/community) is associated with CORE-OM, with adjustment for any participant baseline characteristics that are unbalanced according to follow-up location status (but not adjusting for trial arm). Should this observational analysis indicate that prison location is a statistically significant predictor of CORE-OM, we will proceed to perform a mediation analysis using a structural equation modelling approach, to analyse whether the effect of randomised intervention allocation on 6-month CORE-OM is mediated by location (prison/community) at the same follow-up time.

8 Tables and figures

Examples of tables and figures are set out below.

9 Audit trail

Keep a document of changes to the SAP.

| Date of SAP | SAP version number | Date presented to Trial management group/Trial steering committee | Significant amendments since previous version | Date approved |
| --- | --- | --- | --- | --- |
| 21 May 2018 | 1.1 | 6^th^ June 2018 | N/A |  |
| 14 August 2018 | 1.1.2 | 19/10/2018 | Definition of per protocol for treatment arm confirmed (Section 5.7) |  |
| 19 October | 1.1.3 | 19/10/2018 | Added 12-month analyses (Section 7.2, 7.3, Tables 4–14, 15a, 16, 17) |  |
| 06 December 2018 | 1.1.4 | TBC | Added sensitivity analysis for participants interviewed outside scheduled follow-up window (Section 7.2) |  |
|  | 1.1.5 |  | Post hoc analyses to investigate effect of location at follow-up on CORE-OM and potential mediation effect of randomised allocation on CORE-OM via location at follow-up (Section 7.10) |  |

References

1. International Conference on Harmonisation of Technical Requirements for Registration of Pharmaceuticals for Human Use. Statistical Principles for Clinical Trials. Guideline E9. http://www.ich.org/LOB/media/MEDIA485.pdf

2. Schulz K F, Altman D G, Moher D. CONSORT 2010 Statement: updated guidelines for reporting parallel group randomised trials BMJ 2010; 340 :c332 doi:10.1136/bmj.c332

Figure 1 CONSORT Flow Diagram

**Table 3 Participant demographic characteristics at baseline**

| Characteristic | Engager intervention (N=X) | Usual care (N+X) |
| --- | --- | --- |
| Age (mean (SD), median [range] |  |  |
| Ethnic group n (%):  White English, Welsh, Scottish Irish  White Other  Black British  Black African  Mixed Background  Other Asian Background  Arab  Other |  |  |
| Accommodation n (%)  Stable accommodation:  Own house of flat  House, flat or room rented from housing association of local authority  House, flat or room rented from private landlord  Other |  |  |
| Unstable accommodation:  B&B, hotel, boarding hostel  Hostel or refuge  Homeless  Staying with friend or family, but have own room  Sofa-surfing  Supported accommodation (24hr staffing)  Supported accommodation (not 24hr staffing)  Other |  |  |
| In accommodation dictated by criminal justice system:  Prison  Probation hostel |  |  |
| Educational background n (%):  No qualifications  Basic school level qualifications  A’ level  Degree / HND / HNC / Professional |  |  |
| In education prior to prison:  Full time  Part time  Not in education |  |  |
| Pre-prison employment status n (%) |  |  |
| Employed prior to prison:  Full time paid employment  Part-time paid employment  Full-time self employed  Part-time self employed  Voluntary work |  |  |
| Not working |  |  |
| Other (e.g. retired, carer) |  |  |
| Pre-prison benefits receipt n (%) |  |  |
| Pre-prison income source n (%) |  |  |
| Employment |  |  |
| Benefits |  |  |
| Pre-prison income <£13,500 n (%) |  |  |
| Has current partner n (%) |  |  |
| Has no contact with family n (%) |  |  |
| Has physical health problems n (%) |  |  |
| Has experienced trauma n (%) |  |  |
| Diagnosis of ADHD |  |  |
| Diagnosis of Asperger’s Syndrome or other autistic spectrum disorder |  |  |
| Previous head injury |  |  |
| Standard Assessment of Personality mean (sd) |  |  |
| Alcohol problem (self-report) n (%) |  |  |
| Drug problem (self-report) n (%) |  |  |
| History of self-harm n (%) |  |  |
| Self-harm in last 3 months |  |  |
| Self-harm with suicidal intent in last 3 months |  |  |
| Days in prison on current sentence mean (sd), median (range) |  |  |
| Number of previous imprisonments |  |  |
| 0 |  |  |
| 1 |  |  |
| 2–5 |  |  |
| 6–10 |  |  |
| >10 |  |  |
| Site n (%) |  |  |
| Devon |  |  |
| Manchester |  |  |

Accommodation: Stable accommodation included owning own home or in a house or flat rented from a private landlord or local authority; Unstable accommodation included staying in a B&B, boarding house, hotel, hostel, or refuge, or staying with friends, sofa surfing, or homeless; Requirement of the criminal justice system included being in prison or a probation hostel.

**Table 4 CORE-OM at baseline and follow-up**

|  | Baseline | | 1-month follow-up | | 3-month follow-up | | 6-month follow-up | | | 12-month follow-up | | |
| --- | --- | --- | --- | --- | --- | --- | --- | --- | --- | --- | --- | --- |
| Outcome^a^ | Engager (N=X) | Usual care (N=X) | Engager (N=X) | Usual care (N=X) | Engager (N=X) | Usual care (N=X) | Engager (N=X) | Usual care (N=X) | Between group difference; Engager vs Usual care^b^ | Engager (N=X) | Usual care (N=X) | Between group difference; Engager vs Usual care^b^ |
| **CORE-OM** ITT |  |  |  |  |  |  |  |  |  |  |  |  |
| Per protocol |  |  |  |  |  |  |  |  |  |  |  |  |
| CACE analysis |  |  |  |  |  |  |  |  |  |  |  |  |
| ITT observed and imputed data^c^ |  |  |  |  |  |  |  |  |  |  |  |  |
| **CORE-OM Wellbeing** ITT |  |  |  |  |  |  |  |  |  |  |  |  |
| Per protocol |  |  |  |  |  |  |  |  |  |  |  |  |
| CACE analysis | - | - | - | - |  |  | - | - | - | - |  |  |
| ITT observed and imputed data^c^ | - | - | - | - |  |  | - | - |  |  |  |  |
| **CORE-OM Symptoms**  ITT |  |  |  |  |  |  |  |  |  |  |  |  |
| Per protocol |  |  |  |  |  |  |  |  |  |  |  |  |
| CACE analysis | - | - | - | - |  |  | - | - |  |  |  |  |
| ITT observed and imputed data^c^ | - | - | - | - |  |  | - | - |  |  |  |  |
| **CORE-OM Functioning** ITT |  |  |  |  |  |  |  |  |  |  |  |  |
| Per protocol |  |  |  |  |  |  |  |  |  |  |  |  |
| CACE analysis |  |  |  |  |  |  |  |  |  |  |  |  |
| ITT observed and imputed data^c^ |  |  |  |  |  |  |  |  |  |  |  |  |
| **CORE-OM Risk** ITT |  |  |  |  |  |  |  |  |  |  |  |  |
| Per protocol |  |  |  |  |  |  |  |  |  |  |  |  |
| CACE analysis |  |  |  |  |  |  |  |  |  |  |  |  |
| ITT observed and imputed data^c^ |  |  |  |  |  |  |  |  |  |  |  |  |
| Repeated measures analysis ITT: interaction between treatment group and timepoints: mean difference (95% CI), global p-value | | | | | | | | | | | |  |
| **CORE-OM** |  |  |  |  |  |  |  |  |  |  |  |  |
| **CORE-M Wellbeing** |  |  |  |  |  |  |  |  |  |  |  |  |
| **CORE-OM Symptoms** |  |  |  |  |  |  |  |  |  |  |  |  |
| **CORE-OM Functioning** |  |  |  |  |  |  |  |  |  |  |  |  |
| **CORE-OM Risk** |  |  |  |  |  |  |  |  |  |  |  |  |

ITT Intention to treat. ^a^Outcome is mean (sd), n; unless otherwise stated. ^b^Mean difference (95% confidence interval), *p*-value. Adjusted for site ^c^Data imputed for patients with data unavailable at 12-month follow-up and not known to be deceased.

**Table 5 CORE-10 at baseline and follow-up**

|  | Baseline | | 1-month follow-up | | 3-month follow-up | | 6-month follow-up | | | 12-month follow-up | | |
| --- | --- | --- | --- | --- | --- | --- | --- | --- | --- | --- | --- | --- |
| Outcome^a^ | Engager (N=X) | Usual care (N=X) | Engager (N=X) | Usual care (N=X) | Engager (N=X) | Usual care (N=X) | Engager (N=X) | Usual care (N=X) | Between group difference; Engager vs Usual care^b^ | Engager (N=X) | Usual care (N=X) | Between group difference; Engager vs Usual care^b^ |
| **CORE-10** ITT |  |  |  |  |  |  |  |  |  |  |  |  |
| Per protocol |  |  |  |  |  |  |  |  |  |  |  |  |
| CACE analysis |  |  |  |  |  |  |  |  |  |  |  |  |
| ITT observed and imputed data^c^ |  |  |  |  |  |  |  |  |  |  |  |  |
| Repeated measures analysis ITT: interaction between treatment group and timepoints: mean difference (95% CI), global p-value | | | | | | | | | | | | |
| **CORE-10** |  |  |  |  |  |  |  |  |  |  |  |  |

CACE: Complier average causal effect. ITT Intention to treat. ^a^Outcome is mean (sd), n; unless otherwise stated. ^b^Mean difference (95% confidence interval), *p*-value. Adjusted for site ^c^Data imputed for patients with data unavailable at 12-month follow-up and not known to be deceased.

**Table 6 CAN-FOR at baseline and follow-up**

|  | Baseline | | 3-month follow-up | | 6-month follow-up | | | 12-month follow-up | | |
| --- | --- | --- | --- | --- | --- | --- | --- | --- | --- | --- |
| Outcome^a^ | Engager (N=X) | Usual care (N=X) | Engager (N=X) | Usual care (N=X) | Engager (N=X) | Usual care (N=X) | Between group difference; Engager vs Usual care^b^ | Engager (N=X) | Usual care (N=X) | Between group difference; Engager vs Usual care^b^ |
| **CAN-FOR** ITT |  |  |  |  |  |  |  |  |  |  |
| Per protocol |  |  |  |  |  |  |  |  |  |  |
| CACE analysis |  |  |  |  |  |  |  |  |  |  |
| ITT observed and imputed data^c^ |  |  |  |  |  |  |  |  |  |  |
| Repeated measures analysis ITT: interaction between treatment group and timepoints: mean difference (95% CI), global p-value | | | | | | | | | | |
| **CAN-FOR** |  |  |  |  |  |  |  |  |  |  |

CACE: Complier average causal effect. ITT Intention to treat. ^a^Outcome is mean (sd), n; unless otherwise stated. ^b^Mean difference (95% confidence interval), *p*-value. Adjusted for site ^c^Data imputed for patients with data unavailable at 12-month follow-up and not known to be deceased.

**Table 7 TOP at baseline and follow-up**

|  | Baseline | | 3-month follow-up | | 6-month follow-up | | | 12-month follow-up | | |
| --- | --- | --- | --- | --- | --- | --- | --- | --- | --- | --- |
| Outcome^a^ | Engager (N=X) | Usual care (N=X) | Engager (N=X) | Usual care (N=X) | Engager (N=X) | Usual care (N=X) | Between group difference; Engager vs Usual care^b^ | Engager (N=X) | Usual care (N=X) | Between group difference; Engager vs Usual care^b^ |
| **TOP** ITT |  |  |  |  |  |  |  |  |  |  |
| Per protocol |  |  |  |  |  |  |  |  |  |  |
| CACE analysis |  |  |  |  |  |  |  |  |  |  |
| ITT observed and imputed data^c^ |  |  |  |  |  |  |  |  |  |  |
| Repeated measures analysis ITT: interaction between treatment group and timepoints: mean difference (95% CI), global p-value | | | | | | | | | | |
| **TOP** |  |  |  |  |  |  |  |  |  |  |

CACE: Complier average causal effect. ITT Intention to treat. ^a^Outcome is mean (sd), n; unless otherwise stated. ^b^Mean difference (95% confidence interval), *p*-value. Adjusted for site ^c^Data imputed for patients with data unavailable at 12-month follow-up and not known to be deceased.

**Table 8 European Quality of Life -5 Dimensions-5 Levels (EQ-5D-5L) at baseline and follow-up**

|  | Baseline | | 3-month follow-up | | 6-month follow-up | | | 12-month follow-up | | |
| --- | --- | --- | --- | --- | --- | --- | --- | --- | --- | --- |
| Outcome^a^ | Engager (N=X) | Usual care (N=X) | Engager (N=X) | Usual care (N=X) | Engager (N=X) | Usual care (N=X) | Between group difference; Engager vs Usual care^b^ | Engager (N=X) | Usual care (N=X) | Between group difference; Engager vs Usual care^b^ |
| **EQ-5D-5L** ITT |  |  |  |  |  |  |  |  |  |  |
| Per protocol |  |  |  |  |  |  |  |  |  |  |
| CACE analysis |  |  |  |  |  |  |  |  |  |  |
| ITT observed and imputed data^c^ |  |  |  |  |  |  |  |  |  |  |
| Repeated measures analysis ITT: interaction between treatment group and timepoints: mean difference (95% CI), global p-value | | | | | | | | | | |
| **EQ-5D-5L** |  |  |  |  |  |  |  |  |  |  |

CACE: Complier average causal effect. ITT Intention to treat. ^a^Outcome is mean (sd), n; unless otherwise stated. ^b^Mean difference (95% confidence interval), *p*-value. Adjusted for site ^c^Data imputed for patients with data unavailable at 12-month follow-up and not known to be deceased.

**Table 9 ICE-CAP at baseline and follow-up**

|  | Baseline | | 3-month follow-up | | 6-month follow-up | | | 12-month follow-up | | |
| --- | --- | --- | --- | --- | --- | --- | --- | --- | --- | --- |
| Outcome^a^ | Engager (N=X) | Usual care (N=X) | Engager (N=X) | Usual care (N=X) | Engager (N=X) | Usual care (N=X) | Between group difference; Engager vs Usual care^b^ | Engager (N=X) | Usual care (N=X) | Between group difference; Engager vs Usual care^b^ |
| **ICE-CAP** ITT |  |  |  |  |  |  |  |  |  |  |
| Per protocol |  |  |  |  |  |  |  |  |  |  |
| CACE analysis |  |  |  |  |  |  |  |  |  |  |
| ITT observed and imputed data^c^ |  |  |  |  |  |  |  |  |  |  |
| Repeated measures analysis ITT: interaction between treatment group and timepoints: mean difference (95% CI), global p-value | | | | | | | | | |  |
| **ICE-CAP** |  |  |  |  |  |  |  |  |  |  |

CACE: Complier average causal effect. ITT Intention to treat. ^a^Outcome is mean (sd), n; unless otherwise stated. ^b^Mean difference (95% confidence interval), *p*-value. Adjusted for site ^c^Data imputed for patients with data unavailable at 12-month follow-up and not known to be deceased.

**Table 10 Brief INSPIRE at pre-release and follow-up**

|  | Pre-release | | 3-month follow-up | | 6-month follow-up | | | 12-month follow-up | | |
| --- | --- | --- | --- | --- | --- | --- | --- | --- | --- | --- |
| Outcome^a^ | Engager (N=X) | Usual care (N=X) | Engager (N=X) | Usual care (N=X) | Engager (N=X) | Usual care (N=X) | Between group difference; Engager vs Usual care^b^ | Engager (N=X) | Usual care (N=X) | Between group difference; Engager vs Usual care^b^ |
| **Brief INSPIRE** ITT |  |  |  |  |  |  |  |  |  |  |
| Per protocol |  |  |  |  |  |  |  |  |  |  |
| CACE analysis |  |  |  |  |  |  |  |  |  |  |
| ITT observed and imputed data^c^ |  |  |  |  |  |  |  |  |  |  |
| Repeated measures analysis ITT: interaction between treatment group and timepoints: mean difference (95% CI), global p-value | | | | | | | | | |  |
| **Brief INSPIRE** |  |  |  |  |  |  |  |  |  |  |

CACE: Complier average causal effect. ITT Intention to treat. ^a^Outcome is mean (sd), n; unless otherwise stated. ^b^Mean difference (95% confidence interval), *p*-value. Adjusted for site ^c^Data imputed for patients with data unavailable at 12-month follow-up and not known to be deceased.

**Table 11 Perceived helpfulness of services (Client Service Receipt Inventory) at pre-release and follow-up**

|  | Pre-release | | 3-month follow-up | | 6-month follow-up | | | 12-month follow-up | | |
| --- | --- | --- | --- | --- | --- | --- | --- | --- | --- | --- |
| Outcome^a^ | Engager (N=X) | Usual care (N=X) | Engager (N=X) | Usual care (N=X) | Engager (N=X) | Usual care (N=X) | Between group difference; Engager vs Usual care^b^ | Engager (N=X) | Usual care (N=X) | Between group difference; Engager vs Usual care^b^ |
| **Perceived helpfulness** ITT |  |  |  |  |  |  |  |  |  |  |
| Per protocol |  |  |  |  |  |  |  |  |  |  |
| CACE analysis |  |  |  |  |  |  |  |  |  |  |
| ITT observed and imputed data^c^ |  |  |  |  |  |  |  |  |  |  |
| Repeated measures analysis ITT: interaction between treatment group and timepoints: mean difference (95% CI), global p-value | | | | | | | | | | |
| **Perceived helpfulness** |  |  |  |  |  |  |  |  |  |  |

CACE: Complier average causal effect. ITT Intention to treat. ^a^Outcome is mean (sd), n; unless otherwise stated. ^b^Mean difference (95% confidence interval), *p*-value. Adjusted for site ^c^Data imputed for patients with data unavailable at 12-month follow-up and not known to be deceased.

**Table 12 Leeds Dependence Questionnaire at baseline and follow-up**

|  | Baseline | | 6-month follow-up | | | 12-month follow-up | | |
| --- | --- | --- | --- | --- | --- | --- | --- | --- |
| Outcome^a^ | Engager (N=X) | Usual care (N=X) | Engager (N=X) | Usual care (N=X) | Between group difference; Engager vs Usual care^b^ | Engager (N=X) | Usual care (N=X) | Between group difference; Engager vs Usual care^b^ |
| **LDQ** ITT |  |  |  |  |  |  |  |  |
| Per protocol |  |  |  |  |  |  |  |  |
| CACE analysis |  |  |  |  |  |  |  |  |
| ITT observed and imputed data^c^ |  |  |  |  |  |  |  |  |
| Repeated measures analysis ITT: interaction between treatment group and timepoints: mean difference (95% CI), global p-value | | | | | | | | |
| **LDQ** |  |  |  |  |  |  |  |  |

CACE: Complier average causal effect. ITT Intention to treat. ^a^Outcome is mean (sd), n; unless otherwise stated. ^b^Mean difference (95% confidence interval), *p*-value. Adjusted for site ^c^Data imputed for patients with data unavailable at 12-month follow-up and not known to be deceased.

**Table 13 Intermediate Outcome Measurement Instrument at baseline and follow-up**

|  | Baseline | | 6-month follow-up | | | 12-month follow-up | | |
| --- | --- | --- | --- | --- | --- | --- | --- | --- |
| Outcome^a^ | Engager (N=X) | Usual care (N=X) | Engager (N=X) | Usual care (N=X) | Between group difference; Engager vs Usual care^b^ | Engager (N=X) | Usual care (N=X) | Between group difference; Engager vs Usual care^b^ |
| **IOMI** ITT |  |  |  |  |  |  |  |  |
| Per protocol |  |  |  |  |  |  |  |  |
| CACE analysis |  |  |  |  |  |  |  |  |
| ITT observed and imputed data^c^ |  |  |  |  |  |  |  |  |
| Repeated measures analysis ITT: interaction between treatment group and timepoints: mean difference (95% CI), global p-value | | | | | | | | |
| **IOMI** |  |  |  |  |  |  |  |  |

CACE: Complier average causal effect. ITT Intention to treat. ^a^Outcome is mean (sd), n; unless otherwise stated. ^b^Mean difference (95% confidence interval), *p*-value. Adjusted for site ^c^Data imputed for patients with data unavailable at 12-month follow-up and not known to be deceased.

**Table 14 Social outcomes at baseline and follow-up**

|  | Pre-release | | 6-month follow-up | | | 12-month follow-up | | |
| --- | --- | --- | --- | --- | --- | --- | --- | --- |
| Outcome^a^ | Engager (N=X) | Usual care (N=X) | Engager (N=X) | Usual care (N=X) | Between group difference; Engager vs Usual care^b^ | Engager (N=X) | Usual care (N=X) | Between group difference; Engager vs Usual care^b^ |
| **Housing** ITT |  |  |  |  |  |  |  |  |
| Per protocol |  |  |  |  |  |  |  |  |
| CACE analysis |  |  |  |  |  |  |  |  |
| ITT observed and imputed data^c^ |  |  |  |  |  |  |  |  |
| Repeated measures analysis ITT: interaction between treatment group and timepoints: mean difference (95% CI), global p-value | | | | | | | | |
| **Housing** |  |  |  |  |  |  |  |  |

CACE: Complier average causal effect. ITT Intention to treat. ^a^Outcome is n/N (%); unless otherwise stated. ^b^ Odds ratio (95% confidence interval), *p*-value. Adjusted for site ^c^Data imputed for patients with data unavailable at 12-month follow-up and not known to be deceased.

**Table 15 Reoffending at follow-up**

|  | Pre-release | | 12-month follow-up | | |
| --- | --- | --- | --- | --- | --- |
| Outcome^a^ | Engager (N=X) | Usual care (N=X) | Engager (N=X) | Usual care (N=X) | Between group difference; Engager vs Usual care^b^ |
| **Reoffended** ITT |  |  |  |  |  |
| Per protocol |  |  |  |  |  |
| CACE analysis |  |  |  |  |  |
| ITT observed and imputed data^c^ |  |  |  |  |  |

CACE: Complier average causal effect. ITT Intention to treat. ^a^Outcome is n/N (%); unless otherwise stated. ^b^ Odds ratio (95% confidence interval), *p*-value. Adjusted for site ^c^Data imputed for patients with data unavailable at 12-month follow-up and not known to be deceased.

**Table 15.a Reincarceration and 3, 6 and 12-month follow-up**

|  | 3-month follow-up | | 6-month follow-up | | | 12-month follow-up | | |
| --- | --- | --- | --- | --- | --- | --- | --- | --- |
| Outcome^a^ | Engager (N=X) | Usual care (N=X) | Engager (N=X) | Usual care (N=X) | Between group difference; Engager vs Usual care^a^ | Engager (N=X) | Usual care (N=X) | Between group difference; Engager vs Usual care^a^ |
| In prison at follow-up; n/N (%) |  |  |  |  |  |  |  |  |

^a^ Odds ratio (95% confidence interval), *p*-value. Adjusted for site

**Table 16 CORE-OM at 6- and 12-month follow-up: interactions between intervention status and covariates**

|  | 6-month follow-up | | 12-month follow-up | |
| --- | --- | --- | --- | --- |
| Covariate | Engager vs Usual care: mean difference (95% confidence interval)^a,^ | Global *p*-value | Engager vs Usual care: mean difference (95% confidence interval)^a^, | Global p-value |
| **Site Reference: Devon** |  |  |  |  |
| Manchester |  |  |  |  |
| **Trauma reference: No trauma** |  |  |  |  |
| Sexual trauma |  |  |  |  |
| Relational trauma |  |  |  |  |
| **Personality disorder (SAPAS)** |  |  |  |  |
| **Pre-prison housing: reference: Stable** |  |  |  |  |
| Unstable |  |  |  |  |
| Enforced |  |  |  |  |
| **Alcohol/substance use**  **Reference: No/non-regular use** |  |  |  |  |
| Regular |  |  |  |  |

^a^All analyses are intention to treat. Adjusted for site.

**Table 17 CAN at 6- and 12-month follow-up: interactions between intervention status and covariates**

|  | 6-month follow-up | | 12-month follow-up | |
| --- | --- | --- | --- | --- |
| Covariate | Engager vs Usual care: mean difference (95% confidence interval)^a,^ | Global *p*-value | Engager vs Usual care: mean difference (95% confidence interval)^a,^ | Global *p*-value |
| **Site Reference: Devon** |  |  |  |  |
| Manchester |  |  |  |  |
| **Trauma reference: No trauma** |  |  |  |  |
| Sexual trauma |  |  |  |  |
| Relational trauma |  |  |  |  |
| **Personality disorder (SAPAS)** |  |  |  |  |
| **Pre-prison housing: reference: Stable** |  |  |  |  |
| Unstable |  |  |  |  |
| Enforced |  |  |  |  |
| **Alcohol/substance use**  **Reference: No/non-regular use** |  |  |  |  |
| Regular |  |  |  |  |

^a^All analyses are intention to treat. Adjusted for site.

**Table 18 Re-offending at 12-month follow-up: interactions between intervention status and covariates**

| Covariate | Engager vs Usual care: mean difference (95% confidence interval)^a,^ | Global *p*-value |
| --- | --- | --- |
| **Site Reference: Devon** |  |  |
| Manchester |  |  |
| **Trauma reference: No trauma** |  |  |
| Sexual trauma |  |  |
| Relational trauma |  |  |
| **Personality disorder (SAPAS)** |  |  |
| **Pre-prison housing: reference: Stable** |  |  |
| Unstable |  |  |
| Enforced |  |  |
| **Alcohol/substance use**  **Reference: No/non-regular use** |  |  |
| Regular |  |  |

^a^All analyses are intention to treat. Adjusted for site.

**Table 19 Serious adverse events**

|  | Baseline to 3-month follow-up | | 3- to 6-month follow-up | | 6-to 12-month follow-up | |
| --- | --- | --- | --- | --- | --- | --- |
| Outcome | Engager (N=X) | Usual care (N=X) | Engager (N=X) | Usual care (N=X) | Engager (N=X) | Usual care (N=X) |
| Adverse event^a^; n/N (%) |  |  |  |  |  |  |
| Participants with at least one adverse event; n/N (%) |  |  |  |  |  |  |

^a^Adverse event categories to be confirmed.

1.6 Service Use Data for intervention and usual care

**Table 1: Intervention receipt across both sites**

|  | Southwest  (*n* = 70) | North west  (*n* = 70) | Both Sites  (*n* = 140) |
| --- | --- | --- | --- |
| Received a session in prison, n (%) | 67 (95·7) | 62 (88·6) | 129 (92·1) |
| Number of prison sessions, m (sd, range ) | 6·2 (3·7, 1-17) | 5·2 (4·1, 1-22) | 5·7 (3·9, 1-22) |
| Received a session in community, n (%) | 60 (85·7) | 48 (68·6) | 108 (77·1) |
| Number of community sessions, m (sd, range) | 14·3 (12·4, 1-68) | 11·0 (10·3, 1-46) | 12·9 (11·6, 1-68) |

sd = Standard deviation

**Table 2: Mental health care and contacts by professional/service – Control**

| **Profession** | **Baseline** | **Pre-release** | **Community** |
| --- | --- | --- | --- |
|  | **n=140** | **n=129** | **n=94** |
| **Community mental health nurse** |  |  |  |
| % (n) received | 9% (12) | 5% (7) | 6% (6) |
| Mean (SD) contacts for those receiving | 6.2 (9.1) | 5.6 (9.7) | 16.3 (24.8) |
| **Face-to-face talking therapy NHS** |  |  |  |
| % (n) received | 4% (5) | 0 | 7% (7) |
| Mean (SD) contacts for those receiving | 6.4 (10.0) | 0 | 4.1 (2.7) |
| **Mental Health Clinic (NHS)** |  |  |  |
| % (n) received | 1% (2) | 0 | 1% (1) |
| Mean (SD) contacts for those receiving | 2.5 (2.1) | 0 | 1 (.) |
| **Psychiatrist** |  |  |  |
| % (n) received | 8% (11) | 2% (2) | 3% (3) |
| Mean (SD) contacts for those receiving | 1.7 (1.3) | 1 (0) | 1 (0) |
| **Psychologist** |  |  |  |
| % (n) received | 1% (1) | 1% (1) | 3% (3) |
| Mean (SD) contacts for those receiving | 1 (.) | 3 (.) | 4.7 (6.4) |
| **GP (prison or community)** |  |  |  |
| % (n) received | 31% (44) | 0 | 56% (53) |
| Mean (SD) contacts for those receiving | 2.0 (1.6) | 0 | 5.9 (8.0) |
| **Practice nurse (prison or community)** |  |  |  |
| % (n) received | 20% (28) | 1% (1) | 9% (8) |
| Mean (SD) contacts for those receiving | 4.3 (11.5) | 1 (.) | 45.5 (55.7) |

**Table 3: Mental health care and contacts by professional/service – Intervention**

| **Profession** | **Baseline** | **Pre-release** | **Community** |
| --- | --- | --- | --- |
|  | **n=140** | **n=128** | **n=98** |
| **Community mental health nurse** |  |  |  |
| % received | 4% (6) | 5% (6) | 8% (8) |
| Mean (SD) contacts for those receiving | 2.3 (3.3) | 6.3 (9.7) | 12.1 (35.4) |
| **Face-to-face talking therapy NHS** |  |  |  |
| % received | 6% (8) | 0 | 7% (7) |
| Mean (SD) contacts for those receiving | 10.6 (20.0) | 0 | 9.6 (6.2) |
| **Mental Health Clinic (NHS)** |  |  |  |
| % received | 4% (6) | 0 | 4% (4) |
| Mean (SD) contacts for those receiving | 1 (0) | 0 | 7.5 (9.8) |
| **Psychiatrist** |  |  |  |
| % received | 4% (5) | 0 | 6% (6) |
| Mean (SD) contacts for those receiving | 1.4 (0.5) | 0 | 1.7 (1.2) |
| **Psychologist** |  |  |  |
| % received | 1% (1) | 0 | 3% (3) |
| Mean (SD) contacts for those receiving | 1 (.) | 0 | 1.7 (1.2) |
| **GP (prison or community)** |  |  |  |
| % received | 34% (48) | 0 | 53% (52) |
| Mean (SD) contacts for those receiving | 2.6 (1.6) | 0 | 4.8 (4.4) |
| **Practice nurse (prison or community)** |  |  |  |
| % received | 11% (16) | 0 | 12% (12) |
| Mean (SD) contacts for those receiving | 13.4 (24.4) | 0 | 11.5 (34.2) |

1.7 Intention to treat analyses

**Table 4: Repeated measures analysis (intention to treat using observed data only) for the CORE-OM: interaction between treatment group and time points**

|  | **Time point** | | | | | **Global p value** |
| --- | --- | --- | --- | --- | --- | --- |
|  | **Baseline** | **1-month follow-up** | **3-month follow-up** | **6-month follow-up** | **12-month follow-up** |  |
| **Outcome** |  |  |  |  |  |  |
| **CORE-OM Total** | NA | 2·2 (0·1; 4·3) | 1·7 (-0·3; 3·8) | 2·2 (0·1; 4·2) | 2·0 (-0·4; 4·3) | 0·148 |
| **CORE-OM Wellbeing** | NA | 4·3 (1·3; 7·4) | 3·7 (0·7; 6·7) | 3·1 (0·2; 6·0) | 3·6 (0·2; 6·9) | 0·027 |
| **CORE-OM Symptoms** | NA | 2·4 (-0·3; 5·2) | 1·9 (-0·8; 4·6) | 2·5 (-0·1; 5·1) | 3·4 (0·4; 6·4) | 0·138 |
| **CORE-OM Functioning** | NA | 1·9 (-0·5; 4·2) | 1·0 (-1·2; 3·3) | 2·3 (0·1; 4·5) | 1·3 (-1·2; 3·9) | 0·309 |
| **CORE-OM Risk** | NA | 0·9 (-0·8; 2·7) | 1·8 (0·1; 3·5) | 1·1 (-0·6; 2·8) | -0·6 (-2·5; 1·3) | 0·105 |

NA Not Applicable. Results reported as mean difference (95% confidence interval), p-value. Adjusted for site, baseline covariates (unstable accommodation, trauma (three categories), not working, physical health problem), baseline score.

**Table 5: Secondary outcome measures at baseline and follow-up: intention to treat analyses using observed data only**

|  | **Time point** | | | | | | | | | | | | | |
| --- | --- | --- | --- | --- | --- | --- | --- | --- | --- | --- | --- | --- | --- | --- |
|  | **Baseline** | | **3-month follow-up** | | | **6-month follow-up** | | | | **12-month follow-up** | | | |  |
| Outcome^a^ | Engager (n=140) | Usual care (n=140) | Engager (n=83) | Usual care (n=96) | Engager (n=92) | | Usual care (n=92) | Between group difference; Engager vs Usual care^b^ | Engager (n=67) | | Usual care (n=59) | Between group difference; Engager vs Usual care^b^ |  |  |
| **CAN-FOR** | 11·0 (4·8), 139 | 11·5 (5·5), 140 | 8·2 (5·4), 79 | 7·2 (5·5), 85 | 7·3 (5), 92 | | 6·1 (5·3), 88 | 1·0 (-0·4; 2·4), 0·149 | 6·5 (4·5), 59 | | 5·9 (4·9), 55 | 0·6 (-1·2; 2·4), 0·483 |  |  |
| **TOP alcohol ^c^** | 11·0 (10·5), 103 | 13·2 (10·7), 97 | 15·4 (9·8), 47 | 19·8 (8·7), 52 | 17·2 (8·8), 46 | | 19·2 (8·9), 50 | 0·3 (-3·9; 4·5), 0·883 | 19·2 (9·0), 27 | | 18·1 (8·8), 35 | 4·4 (-0·9; 9·8), 0·103 |  |  |
| **TOP opiates ^c^** | 5·6 (9·4), 48 | 5·1 (9·7), 45 | 10·4 (10·7), 16 | 10·9 (10·1), 14 | 18·2 (8·9), 19 | | 21·4 (8·3), 14 | -3·5 (-10·0; 3·0), 0·270 | 18·3 (5·0), 7 | | 14·2 (10·1), 6 | 1·8 (-22·7; 26·3), 0·829 |  |  |
| **EQ-5D-5L** | 0·679 (0·234), 140 | 0·657 (0·225), 140 | 0·705 (0·246), 80 | 0·685 (0·274), 86 | 0·692 (0·241), 92 | | 0·719 (0·251), 88 | -0·042 (-0·118; 0·034), 0·281 | 0·771 (0·211), 59 | | 0·706 (0·265), 56 | 0·079 (-0·051; 0·208), 0·230 |  |  |
| **EQ-5D Visual Analogue Scale (0–100)** | 55 (22), 73 | 56 (21), 61 | 61 (21), 37 | 67 (23), 31 | 59 (23), 44 | | 59 (23), 40 | 0 (14; 14), 0·996 | 66 (22), 28 | | 64 (27), 30 | -4 (-23; 14), 0·629 |  |  |
| **ICECAP-A** | 0·613 (0·221), 140 | 0·613 (0·226), 139 | 0·634 (0·222), 80 | 0·658 (0·254), 86 | 0·656 (0·21), 92 | | 0·708 (0·233), 88 | -0·053 (-0·114; 0·008), 0·087 | 0·717 (0·233), 60 | | 0·728 (0·231), 56 | -0·030 (-0·122; 0·062), 0·519 |  |  |
| **Brief INSPIRE** | 47 (28), 113 | 37 (29), 108 | 45 (31), 76 | 40 (31), 83 | 43 (28), 91 | | 41 (30), 86 | 0 (-9; 9), 0·948 | 49 (26), 61 | | 41 (26), 55 | 4 (-7; 15), 0·505 |  |  |
| **LDQ** | 16·8 (10·3), 139 | 15·4 (10·6), 140 | NA | NA | 6·6 (8·7), 92 | | 4·5 (6·7), 88 | 1·7 (-0·6; 3·9), 0·141 | 4·6 (7·1), 59 | | 6·6 (7·6), 56 | -2·2 (-5·1; 0·7), 0·136 |  |  |
| **IOMI** | 67 (12), 137 | 66 (11), 137 | NA | NA | 71 (9), 88 | | 73 (12), 85 | -2 (-5; 1), 0·217 | 75 (12), 59 | | 72 (13), 55 | 2 (-2; 7), 0·309 |  |  |

^a^ Outcome is mean (standard deviation), n. ^b^ Mean difference (95% confidence interval), p-value. Adjusted for site, baseline covariates (unstable accommodation, trauma (three categories), not working, physical health problem), baseline score. ^c^ days abstinent out of 28, n; unless otherwise stated, CAN-FOR = Camberwell Assessment of Need – Forensic Version, TOP = Treatment Outcomes Profile (TOP), LDQ = Leeds Dependence Questionnaire; ICE-CAP-A = ICEpop CAPability; IOMI = Intermediate Outcomes Measurement Instrument. NA – Not applicable LDQ and IOMI not collected at 3 months.

**Table 6: Accommodation at baseline and follow-up: intention to treat analysis using observed data only**

|  | Baseline | | 6-month follow-up | | | 12-month follow-up | | |
| --- | --- | --- | --- | --- | --- | --- | --- | --- |
| Outcome | Engager (N=140) | Usual care (N=140) | Engager (N=92) | Usual care (N=92) | Between group difference; Engager vs Usual care^b^ | Engager (N=67) | Usual care (N=59) | Between group difference; Engager vs Usual care^b^ |
| **Accommodation status**^2^ |  |  | N=92 | N=88 |  | N=61 | N=56 |  |
| Stable | 56 (40) | 73 (52) | 27 (29) | 37 (42) | - | 19 (31) | 30 (54) | - |
| Unstable | 76 (54) | 58 (41) | 42 (46) | 35 (40) | - | 29 (48) | 19 (34) | - |
| Enforced | 8 (6) | 8 (6) | 23 (25) | 16 (18) | - | 13 (21) | 7 (13) | - |
| Other | 0 (0) | 1 (1) | 0 (0) | 0 (0) | - | 0 (0) | 0 (0) | - |
| **Accommodation status: unstable vs stable** | - | - | - | - | 1·31 (0·58; 2·95), 0·518 | - | - | 1·72 (0·57; 5·18), 0·338 |

^a^ Outcome is n (%); ^b^ Odds ratio (95% confidence interval), p-value. Adjusted for site, baseline covariates (unstable accommodation, trauma (three categories), not working, physical health problem). ^c^ Accommodation: stable accommodation: own home, house/flat rented from a private landlord or local authority; unstable accommodation: B&B, boarding house, hotel, hostel, refuge, staying with friends, sofa surfing, homeless; enforced accommodation: prison, probation hostel.

**Table 7: Repeated measures analysis for all secondary outcome (intention to treat using observed data only): interaction between treatment group and time points**

|  | **Time point** | | | | **Global p-value** |  |
| --- | --- | --- | --- | --- | --- | --- |
|  | **Baseline** | **3-month follow-up** | **6-month follow-up** | **12-month follow-up** |  |  |
| **CAN-FOR** | NA | 1·0 (-0·4; 2·4) | 1·1 (-0·3; 2·5) | 1·4 (-0·2; 3·1) | 0·228 |  |
| **TOP alcohol** | NA | -1·2 (-5·0; 2·7) | 1·6 (-2·3; 5·5) | 2·6 (-2·0; 7·3) | 0·432 |  |
| **TOP opiates** | NA | -1·1 (-7·7; 5·4) | -3·0 (-9·4; 3·4) | 0·7 (-8·5; 10·0) | 0·811 |  |
| **EQ-5D-5L** | NA | 0·016 (-0·060; 0·092) | -0·040 (-0·115; 0·035) | 0·023 (-0·066; 0·111) | 0·484 |  |
| **EQ-5D Visual Analogue Scale (0–100)** | NA | -1 (-13; 10) | 4 (-6; 15) | 3 (-9; 16) | 0·779 |  |
| **ICECAP-A** | NA | -0·002 (-0·068; 0·065) | -0·035 (-0·100; 0·029) | -0·018 (-0·095; 0·058) | 0·712 |  |
| **Brief INSPIRE** | NA | -4 (-13; 5) | -7 (-16; 2) | -1 (-12; 9) | 0·445 |  |
| **LDQ** | NA | NA | -0·1 (-2·8; 2·6) | -3·4 (-6·6; -0·2) | 0·088 |  |
| **IOMI** | NA | NA | -2 (-6; 1) | 0 (-3; 4) | 0·271 |  |

NA = Not Applicable; CAN-FOR = Camberwell Assessment of Need – Forensic Version, TOP = Treatment Outcomes Profile (TOP), LDQ = Leeds Dependence Questionnaire; ICE-CAP-A = ICEpop CAPability; IOMI = Intermediate Outcomes Measurement Instrument. Adjusted for site, baseline covariates (unstable accommodation, trauma (three categories), not working, physical health problem), baseline score.

1.8 Per protocol analyses

Per protocol was defined as at least two sessions in prison and eight sessions in the community.

**Table 8**

|  | **Baseline** | | **1-month follow-up** | | **3-month follow-up** | | **6-month follow-up** | | | **12-month follow-up** | | |
| --- | --- | --- | --- | --- | --- | --- | --- | --- | --- | --- | --- | --- |
| **Outcome^a^** | **Engager** | **Usual care** | **Engager** | **Usual care** | **Engager** | **Usual care** | **Engager** | **Usual care** | **Between group difference; Engager vs Usual care^b^** | **Engager** | **Usual care** | **Between group difference; Engager vs Usual care^b^** |
| **CORE-OM Total** | 14·5 (5·6), 62 | 16·9 (6·2), 140 | 12·4 (8·1), 47 | 12·6 (8·4), 76 | 13·5 (7·2), 45 | 12·8 (7·8), 88 | 12·5 (6·8), 53 | 11·9 (7·7), 90 | 0·9 (-1·6; 3·5), 0·473 | 11·4 (6·8), 37 | 12 (8·9), 58 | -0·9 (-4·5; 2·6), 0·605 |
| **CORE-OM Wellbeing** | 16·0 (9·9), 62 | 19·6 (8·3), 139 | 15·1 (10·9), 45 | 14·6 (10·6), 76 | 15·9 (9·2), 44 | 14·1 (10·5), 87 | 14·9 (9.8), 52 | 14·0 (10), 89 | 1·4 (-2.0; 4·8), 0·428 |  |  | 0·6 (-4·1; 5·3), 0·804 |
| **CORE-OM Symptoms** | 18·5 (7·7), 62 | 21·4 (8·6), 140 | 15·1 (10·6), 47 | 14·9 (10·3), 76 | 16·7 (10), 45 | 15·6 (9·7), 88 | 15 (8·4), 53 | 14·4 (9·5), 90 | 1·0 (-2·1; 4·2), 0·524 | 14.3 (9.1), 37 | 14.0 (10.9), 58 | 0.2 (-4.1; 4.6), 0.910 |
| **CORE-OM Functioning** | 16 (6), 62 | 18·2 (6·7), 140 | 13·6 (8·5), 47 | 14·1 (9·2), 76 | 14·4 (7·4), 45 | 14·5 (8·5), 88 | 13·8 (7·5), 53 | 12·9 (8·2), 90 | 1·2 (-1·6; 4·0), 0·406 | 12.5 (7.9), 37 | 13.4 (9.1), 58 | -1.4 (-5.3; 2.5), 0.475 |
| **CORE-OM Risk** | 2·3 (3·7), 62 | 3·7 (5·3), 137 | 2·7 (4·8), 47 | 3·7 (5·8), 74 | 3·9 (4·9), 45 | 2·9 (5·3), 85 | 3·3 (5·2), 53 | 3·2 (5·3), 88 | 0·2 (-1·7; 2·1), 0·866 | 2.5 (4.2), 37 | 4.9 (6.7), 58 | -3.2 (-5.7; -0.6), 0.016 |
| **CAN-FOR** | 11·8 (4·7), 62 | 11·5 (5·5), 140 | N/A | N/A | 8·8 (5·2), 44 | 7·2 (5·5), 85 | 7·5 (5·1), 53 | 6·1 (5·3), 88 | 0·9 (-0·8; 2·6), 0·317 | 7.6 (4.7), 31 | 5.9 (4.9), 55 | 1.2 (-0.9; 3.4), 0.262 |
| **TOP alcohol** | 7·4 (9·7), 43 | 13·2 (10·7), 97 | N/A | N/A | 16·8 (9·4), 24 | 19·8 (8·7), 52 | 15·9 (9·4), 27 | 19·2 (8·9), 50 | -0·7 (-6·0; 4·7), 0·805 | 19.7 (9), 15 | 18.1 (8.8), 35 | 4.3 (-3.1; 11.6), 0.245 |
| **TOP opiates** | 3·5 (7·3), 23 | 5·1 (9·7), 45 | N/A | N/A | 8·5 (11·6), 10 | 10·9 (10·1), 14 | 18·7 (7·5), 10 | 21·4 (8.3), 14 | - | 17.0 (4.5), 4 | 14.2 (10.1), 6 | - |
| **EQ-5D-5L** | 0·683 (0·233), 62 | 0·657 (0·225), 140 | N/A | N/A | 0·728 (0·219), 45 | 0·685 (0·274), 86 | 0·718 (0·181), 53 | 0·719 (0·251), 88 | -0·024 (-0·109; 0·062), 0·585 | 0.769 (0.193), 32 | 0.706 (0.265), 56 | 0.079 (-0.051; 0.208), 0.230 |
| **EQ-5D Health state (0–100)** | 52 (19), 23 | 56 (21), 61 | N/A | N/A | 63 (22), 18 | 67 (23), 31 | 58 (24), 19 | 59 (23), 40 | 0 (-18; 18), 0·982 | 63 (23), 12 | 64 (27), 30 | -8 (-41; 25), 0.616 |
| **ICE-CAP** | 0·599 (0·201), 62 | 0·613 (0·226), 139 | N/A | N/A | 0·632 (0·234), 45 | 0·658 (0·254), 86 | 0·656 (0·205), 53 | 0·708 (0·233), 88 | -0·050 (-0·124; 0·025), 0·190 | 0.677 (0.246), 32 | 0.728 (0.231), 56 | -0.061 (-0.174; 0.052), 0.284 |
| **Brief INSPIRE** | 47 (26), 52 | 37 (29), 108 | N/A | N/A | 47 (33), 41 | 40 (31), 83 | 49 (28), 52 | 41 (30), 86 | 3 (-8; 13), 0·595 | 53 (26), 33 | 41 (26), 55 | 8 (-5; 21), 0.224 |
| **LDQ** | 20·1 (9·7), 62 | 15·4 (10·6), 140 | N/A | N/A | N/A | N/A | 7·5 (8·7), 53 | 4·5 (6·7), 88 | 1·8 (-0·8; 4·4), 0·163 | 4.9 (6.5), 31 | 6.6 (7.6), 56 | -2.9 (-6.2; 0.5), 0.091 |
| **IOMI** | 66 (11), 60 | 66 (11), 137 | N/A | N/A | N/A | N/A | 71 (10), 49 | 73 (12), 85 | -2 (-6; 2), 0·399 | 75 (13), 31 | 72 (13), 55 | 4 (-2; 10), 0.188 |

**^a^ Outcome is mean (sd), n; unless otherwise stated. ^b^ Mean difference (95% confidence interval), p-value. Adjusted for site, baseline covariates (unstable accommodation, trauma (three categories), not working, physical health problem). CAN-FOR = Camberwell Assessment of Need – Forensic Version, TOP = Treatment Outcomes Profile (TOP), LDQ = Leeds Dependence Questionnaire; ICE-CAP-A = ICEpop CAPability; IOMI = Intermediate Outcomes Measurement Instrument; NA Not Applicable**

**Table 9: Per protocol analysis for accommodation**

|  | **Baseline (before prison)** | | **6-month follow-up** | | | **12-month follow-up** | | |
| --- | --- | --- | --- | --- | --- | --- | --- | --- |
| **Outcome^a^** | **Engager** | **Usual care** | **Engager** | **Usual care** | **Between group difference; Engager vs Usual care^b^** | **Engager** | **Usual care** | **Between group difference; Engager vs Usual care^b^** |
| **Accommodation^c^** | N=62 | N=140 | N=53 | N=88 |  | N=33 | N=56 |  |
| Stable | 22 (35) | 73 (52) | 14 (26) | 37 (42) | - | 9 (27) | 30 (54) | - |
| Unstable | 36 (58) | 58 (41) | 25 (47) | 35 (40) | - | 17 (52) | 19 (34) | - |
| Enforced | 4 (6) | 8 (6) | 14 (26) | 16 (18) | - | 7 (21) | 7 (13) | - |
| Other | 0 (0) | 1 (1) | 0 (0) | 0 (0) | - | 0 (0) | 0 (0) | - |
| **Accommodation: unstable vs stable** | - | - | - | - | 1.32 (0.50; 3.46), 0.577 | - | - | 3.47 (0.88; 13.69), 0.076 |

**^a^ Outcome is n (%) ^b^Odds ratio (95% confidence interval), p-value. Adjusted for site, baseline covariates (unstable accommodation, trauma (three categories), not working, physical health problem). Includes only participants in the community at follow-up. ^c^ Accommodation: stable accommodation: own home, house/flat rented from a private landlord or local authority; unstable accommodation: B&B, boarding house, hotel, hostel, refuge, staying with friends, sofa surfing, homeless; enforced accommodation: prison, probation hostel.**

1.9 CACE and interactional analyses

**Table 10: CACE analysis for primary and all secondary outcomes**

|  | **6-month follow-up** | **12-month follow-up** |
| --- | --- | --- |
|  | Between group difference; Engager vs Usual care2 | Between group difference; Engager vs Usual care2 |
| **CORE-OM Total** | 1·6 (-0·7; 3·9), 0·181 | -1·2 (-4·6; 2·1), 0·468 |
| **CORE-OM Wellbeing** | 1·6 (-3·3; 6·5), 0·517 | 0·0 (-6·1; 6·0), 0·993 |
| **CORE-OM Symptoms** | 2·1 (-2·2; 6·5), 0·341 | 1·0 (-4·8; 6·9), 0·726 |
| **CORE-OM Functioning** | 2·4 (-1·5; 6·4), 0·231 | -1·6 (-7·0; 3·7), 0·543 |
| **CORE-OM Risk** | 0·2 (-2·4; 2·7), 0·908 | -4·4 (-8·0; -0·8); 0·017 |
| **CAN-FOR** | 1·7 (-5·9; 4·1), 0·143 | 1·1 (-1·9; 4·1), 0·459 |
| **TOP alcohol** | 0·6 (-6·5; 7·6), 0·877 | 8·4 (-0·9; 17·8), 0·076 |
| **TOP opiates** | - | - |
| **EQ-5D-5L** | -0·072 (-0·203; 0·059), 0·281 | 0·102 (-0·085; 0·289), 0·285 |
| **EQ-5D Health state (0–100)** | 0 (-32; -32), 0·996 | -15 (-64; 34), 0·554 |
| **ICE-CAP** | -0·071 (-0·202; 0·060), 0·286 | 0·095 (-0·091; 0·282), 0·316 |
| **Brief INSPIRE** | -1 (-15; 14), 0·947 | 7 (-12; 25), 0·479 |
| **LDQ** | 2·9 (-0·9; 6·8), 0·133 | -4·0 (-9·0; 1·1), 0·123 |
| **IOMI** | -3 (-9; 2), 0·206 | 4 (-4; 13), ·288 |

**Reported as mean difference (95% confidence interval), p-value. Adjusted for site, baseline covariates (unstable accommodation, trauma (three categories), not working, physical health problem), baseline score.**

**Table 11: CORE-OM total score at six- and 12-month follow-up: interactions between intervention status and covariates**

|  | **6-month follow-up** | | **12-month follow-up** | |
| --- | --- | --- | --- | --- |
| **Covariate** | **Engager vs Usual care: mean difference (95% confidence interval)^1^** | **Global *p*-value** | **Engager vs Usual care: mean difference (95% confidence interval)^1^** | **Global *p*-value** |
| **Site**  **reference: Devon** |  |  |  |  |
| Manchester | -1·3 (-5·3; 2·8) | 0·540 | 2·0 (-3·4; 7·4) | 0·464 |
| **Trauma**  **reference: sexual trauma** |  |  |  |  |
| Relational trauma | -5·0 (-10·2; 0·2) | 0·114 | -1·5 (-8·7; 5·8) | 0·850 |
| No/other trauma | -5·9 (-12·1; 0·3) |  | -2·4 (-10·8; 6·0) |  |
| **Personality disorder (SAPAS)** | -0·2 (-1·4; 1·0) | 0·712 | -0·3 (-1·9; 1·4) | 0·731 |
| **Pre-prison housing reference: stable** |  |  |  |  |
| Unstable/enforced | -0·3 (-4·5; 3·9) | 0·882 | -3·0 (-8·5; 2·5) | 0·277 |
| **Alcohol use problem**  **reference: no problem** |  |  |  |  |
| Problem | -1·1 (-5·2; 3·1) | 0·610 | -5·8 (-11·2; -0·5) | 0·032 |
| **Substance use problem**  **reference: no problem** |  |  |  | 1 |
| Problem | -0·3 (-4·5; 3·9) | 0·890 | -5·0 (-10·5; 0·4) | 0·071 |

**^1^All analyses are intention to treat. Adjusted for site, baseline covariates (unstable accommodation, trauma (three categories), not working, physical health problem), baseline score.**

**Table 12. CAN-FOR at six- and 12-month follow-up: interactions between intervention status and covariates**

|  | **6-month follow-up** | | **12-month follow-up** | |
| --- | --- | --- | --- | --- |
| **Covariate** | **Engager vs Usual care: mean difference (95% confidence interval)^1^** | **Global *p*-value** | **Engager vs Usual care: mean difference (95% confidence interval)^1^** | **Global *p*-value** |
| **Site**  **reference: Devon** |  |  |  |  |
| Manchester | 0·5 (-2·2; 3·1) | 0·727 | -1·6 (-5·0; 1·7) | 0·328 |
| **Trauma**  **reference: sexual trauma** |  |  |  |  |
| Relational trauma | 0·6 (-2·9; 4·1) | 0·687 | 2·1 (-2·4; 6·6) | 0·627 |
| No/other trauma | 1·7 (-2·4; 5·8) |  | 1·2 (-4·1; 6·4) |  |
| **Personality disorder (SAPAS)** | 0·4 (-0·4; 1·2) | 0·351 | 0·0 (-1·0; 1·1) | 0·944 |
| **Pre-prison housing reference: stable** |  |  |  |  |
| Unstable/enforced | 0·2 (-2·5; 2·9) | 0·877 | -0·9 (-4·3; 2·4) | 0·587 |
| **Alcohol use problem**  **reference: no problem** |  |  |  |  |
| Problem | 1·7 (-1·0; 4·4) | 0·215 | 0·2 (-3·2; 3·5) | 0·928 |
| **Substance use problem**  **reference: no problem** |  |  |  |  |
| Problem | -1·1 (-3·9; 1·6) | 0·409 | -2·7 (-6·0; 0·7) | 0·120 |

**^1^All analyses are intention to treat. Adjusted for site, baseline covariates (unstable accommodation, trauma (three categories), not working, physical health problem), baseline score.**
